# Atorvastatin-Loaded Dissolving Microarray Patches for Long-Acting Microdepot Delivery: Comparison of Nanoparticle and Microparticle Drug Formulations

**DOI:** 10.1021/acsami.4c05517

**Published:** 2024-10-02

**Authors:** Yara A. Naser, Lalitkumar K. Vora, Ismaiel A. Tekko, Ke Peng, Fabiana Volpe-Zanutto, Brett Greer, Alejandro Paredes, Helen O. McCarthy, Ryan F. Donnelly

**Affiliations:** † School of Pharmacy, Queen’s University Belfast, Medical Biology Centre, 97 Lisburn Road, Belfast BT9 7BL, Northern Ireland, U.K.; ‡ Department of Pharmaceutics and Pharmaceutical Technology, Faculty of Pharmacy, Aleppo University, Aleppo 00 963, Syria; § Institute for Global Food Security, School of Biological Science, Queen’s University Belfast, 19 Chlorine Gardens, Belfast BT9 5DL, Northern Ireland, U.K.; ⊥ School of Biomedical Sciences, Ulster University, Cromore Road, Coleraine BT52 1SA, Northern Ireland, U.K.

**Keywords:** Microparticles, Nanoparticles, Dissolving microarray patch, Transdermal drug delivery, Microneedles, Long-acting depot, Hyperlipidemia, Atorvastatin

## Abstract

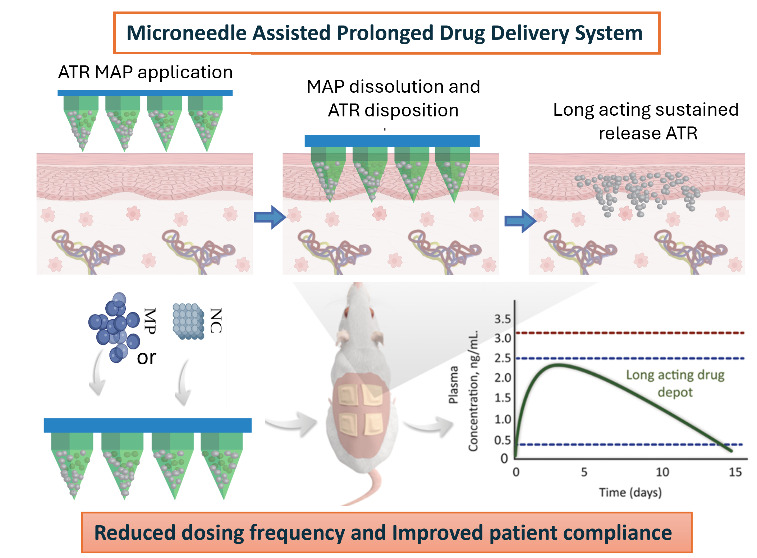

The increasing popularity of prolonged-release dosage forms, owing to their ability to provide continuous drug release after administration, has significantly improved patient compliance and overall quality of life. However, achieving prolonged release beyond 24 h frequently requires the use of invasive methods, including injections or implants, which may prove challenging for people suffering from needle phobia. This study introduces atorvastatin (ATR) microparticles (MPs) or nanocrystal (NCs) dissolving microarray patches (D-MAPs) as a noninvasive alternative for intradermal drug delivery over a two-week period for the management of hyperlipidemia. The MP-loaded D-MAPs exhibited an average drug loading of 5.15 ± 0.4 mg of ATR per patch, surpassing the 2.4 ± 0.11 mg/patch observed with NC-loaded D-MAPs. Skin deposition studies demonstrated the superior performance of MP D-MAPs, which delivered 2.0 ± 0.33 mg of ATR per 0.75 cm^2^ patch within 24 h, representing 38.76% of the initial amount of drug loaded. In contrast, NC D-MAPs delivered approximately 0.89 ± 0.12 mg of ATR per 0.75 cm^2^ patch at 24 h, equivalent to 38.42 ± 5.13% of the initial ATR loaded. Due to their favorable results, MP D-MAPs were chosen for an *in vivo* study using Sprague–Dawley rats. The findings demonstrated the capacity of D-MAPs to deliver and attain therapeutically relevant ATR concentrations (>20 ng/mL) for 14 days after a single 24-h application. This study is the first to successfully demonstrate the long-acting transdermal delivery of ATR using MP-loaded D-MAPs after a 24-h single-dose application. The innovative D-MAP system, particularly when loaded with MP, arises as a promising, minimally invasive, long-acting substitute for ATR delivery. This technology has the potential to improve patient compliance and therapeutic outcomes while also significantly advancing the field of transdermal drug delivery.

## 1 Introduction

Based on the World Health Organization statistics in 2021, cardiovascular disease (CVD) is the leading cause of death worldwide, accounting for 32% of all global deaths in 2019.^2^ Hyperlipidemia is a critical risk factor that leads to the development and progression of atherosclerotic diseases and is considered a major cause of CVD.^3^ Hyperlipidemia is defined as an increase in the concentration of fasting total cholesterol in the body.^4^ The risk of the development of atherosclerosis and CVD increases as the levels of total serum cholesterol or low-density lipoprotein (LDL) cholesterol increase.^5^ Atorvastatin (ATR), a 3-hydroxy-3-methylglutaryl coenzyme A (HMG-CoA) reductase inhibitor for controlling hyperlipidemia, is administered orally at doses ranging from 10 to 80 mg/day to control hyperlipidemia.^5,6^ As a class II drug according to the BCS classification, ATR faces challenges due to its low aqueous solubility and high permeability.^7−9^ The use of particle size reduction, a common method to enhance solubility, is thereby explored in this manuscript. Reducing the particle size to micron or submicron levels is considered an efficient way to increase the drug surface area, dissolution rate, solubility, and bioavailability.^10,11^ Nanocrystals (NCs) were chosen for their nanometer-sized crystalline structure, which was derived from the therapeutic agent itself and surrounded by a thin layer of stabilizer, ensuring high drug content and inherent stability.^12^

Compared to traditional methods such as oral or hypodermic injections, transdermal drug delivery (TDD) is an attractive option.^13^ This is primarily because the skin is the largest and most accessible surface area in the human body.^14,15^ However, the outermost skin layer, the *stratum corneum* (SC), poses a significant barrier to passive drug delivery.^16−18^ Various techniques have been explored to overcome the SC barrier, with microarray patches (MAPs) being one of the most promising approaches. MAPs are micron-sized minimally invasive devices capable of bypassing SC to deliver drug products across the skin without causing any pain or bleeding.^19−21^ Therefore, they can facilitate the transdermal and intradermal delivery of various drugs and vaccines.^15,22,23^

Dissolving microarray patches (D-MAPs), particularly, is a type of MAPs that is vastly used for transdermal drug delivery of a vast range of drugs and therapeutic agents.^13,19,24−30^ Composed of dissolving biocompatible polymers, D-MAPs start to dissolve upon their insertion into the skin, thus liberating their drug content and facilitating their absorption into the dermal microcirculation.^24,31−34^ This is critical for reducing the possibility of needle reinsertion errors and consequently improving their safety profile. They are regarded as self-disabling since they entirely disintegrate in the skin and do not require specialist disposal.^35^

Nanoparticles have been extensively used in the delivery of many conventional drugs, proteins, vaccines and nucleotides.^12^ They can be formulated from a wide variety of materials, including sugars, lipids, degradable or nondegradable polymers, metals and organic or inorganic compounds.^36^ In this study, NCs were chosen due to their high drug content, which could reach approximately 100%.^37^ Additionally, they are considered to be inherently stable, with the requirement of adding small amounts of stabilizers for their production.^37^ In this manuscript, NCs were prepared using a laboratory-scale wet bead-milling technique, chosen for its simplicity, directness, and reproducibility.^35^

The literature highlights the use of D-MAPs as a platform for transdermal delivery, involving microparticles,^24,26^ nanoparticles^28,37−39^ or a mixture of both^40^ aimed at targeted drug delivery^38^ or prolonged effects.^24^ D-MAPs are typically made from cost-effective polymers with excellent safety profiles. D-MAPs in this work were fabricated using low-molecular-weight PVA (9–10 kDa) and PVP (58 kDa). This research marks the first successful application of D-MAPs for the efficient and sustained transdermal delivery of the hydrophobic drug atorvastatin (ATR). In an *in vivo* study utilizing Sprague–Dawley rats, this system demonstrated its versatility by achieving extended delivery of hydrophobic ATR over a 2-week period, with only a single application lasting 24 h. This innovative approach holds promise as a minimally invasive, long-acting alternative for ATR delivery, potentially improving patient adherence to treatment plans and consequently enhancing therapeutic outcomes and overall quality of life.

## 2 Materials and Methods

### 2.1 Materials

Atorvastatin hemicalcium trihydrate was obtained from Cangzhou Enke Pharma-tech Co., Limited, Hebei Province, China. Poly(vinylpyrrolidone) (PVP), with a MW of 58 kDa, sold under the product brand name Plasdone K-29/32 was acquired from Ashland, Kidderminster, UK. Poly(vinyl) alcohol (PVA), MW 9,000–10,000, methanol, and acetonitrile for high-performance liquid chromatography (HPLC), phosphate-buffered saline (PBS) pH 7.4 tablets, and sodium lauryl sulfate (SLS) were all purchased from Sigma–Aldrich, Dorset, UK. All other chemicals and compounds used were of analytical reagent grade

### 2.2 Fabrication of Microparticle (MP)-Loaded D-MAPs

#### 2.2.1 Fabrication of ATR MP-Loaded D-MAPs (MP D-MAPs)

D-MAPs were produced utilizing a bilayer casting technique, as previously reported, to reduce ATR waste. Herein, the high-density molds used in the preparation of ATR D-MAPs featured 600 pyramidal needles within a 0.75 cm^2^ area, each with a base of 300 × 300 μm, a height of 750 μm, and an interspacing of 50 μm. Two distinct layers were prepared individually: the ATR-containing layer (first layer) and the drug-free base plate (second layer). For the first layer, a polymer solution comprising 20% w/w polyvinyl(alcohol) (PVA, MW 9–10 kDa) in deionized water was hydrolyzed at 90 °C for 24 h. In addition, a 20% w/w solution of polyvinylpyrrolidone (PVP, 58 kDa) in deionized water was prepared and sonicated for 24 h to produce a transparent solution. The first layer contained 40% w/w ATR powder, 30% w/w PVA (9–10 kDa), and 30% w/w PVP (58 kDa). This mixture was thoroughly mixed using a dual asymmetric centrifugation device (Speedmixer, Hauschild Engineering, Harnm, Germany) at 3,500 rpm for 5 min. Positive pressure pipettes were used to equally distribute aliquots (50 μL) of the formulation into microneedle (MN) cavities in the molds. The molds were then placed in a pressure chamber at 5 bar for 3 min before being removed and scraped to eliminate any excess formulation. They were then reintroduced to the pressure chamber for 30 min at 5 bar. Two drying periods were tested: 4 h to allow for the casting of both layers on the same day, and 24 h, both at room temperature.

**Figure 1:**
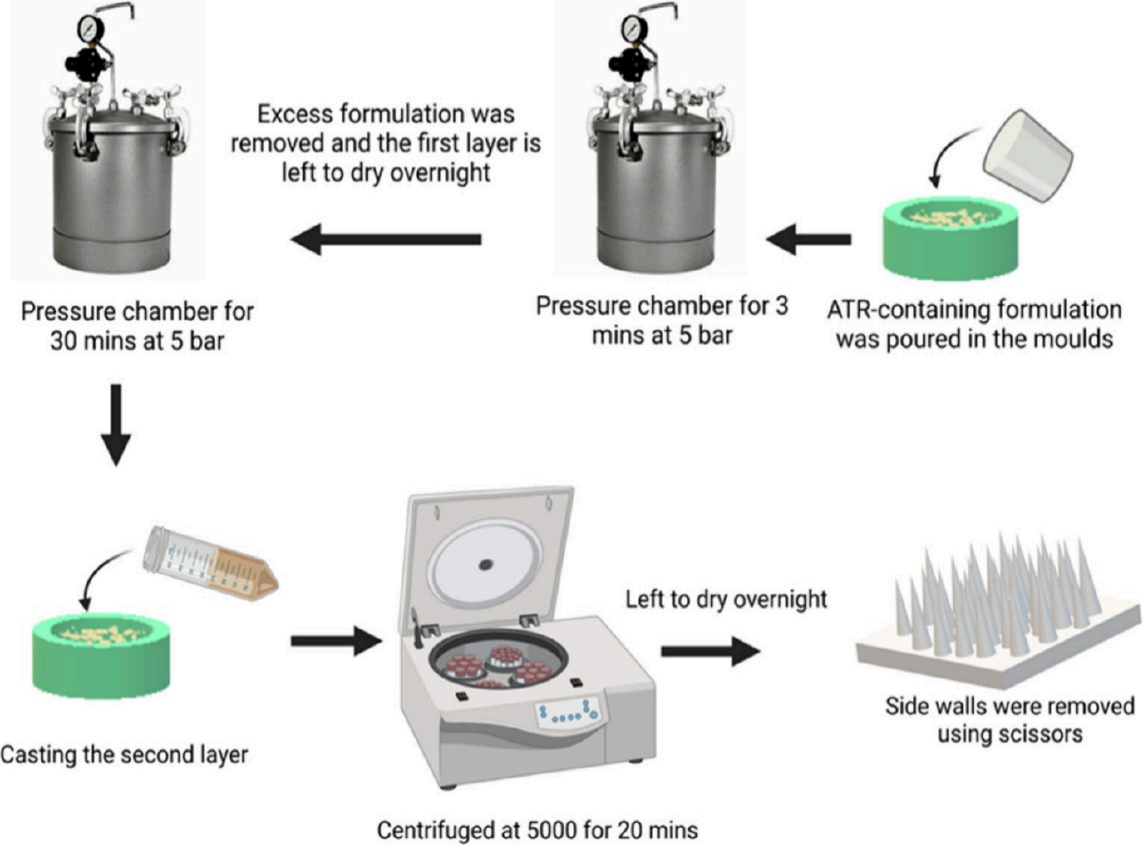
Schematic representation of the steps required for the preparation of MP D-MAPs

To produce the drug-free base plate (second layer), 30% w/w PVP (58 kDa), 1.5% w/w glycerol, and deionized water up to 100% w/w were combined in a 50 mL Falcon tube. The mixture was sonicated overnight and subsequently centrifuged to obtain a clear homogeneous solution. Afterward, aliquots of 500 mg of the second layer formulation were cast on top of the first layer. After centrifugation at 5,000 rpm for 20 min, the molds were allowed to dry for 24 h at room temperature. The D-MAPs were gently removed from the molds, and their sidewalls were trimmed using scissors. Figure 1 provides an overview of the steps involved in mold fabrication and casting both layers of D-MAPs.

#### 2.2.2 Particle Size Determination of the Crude ATR Powder and the D-MAPs Formulation

A Mastersizer 3000 laser diffraction particle size analyzer (Malvern Analytical, Malvern, Worcestershire, United Kingdom) was used for the analysis of the particle size distribution (PSD) for both the first layer formulation (containing ATR, PVA, and PVP with water) and the crude ATR powder. Initially, the crude ATR powder was dispersed in a 10 mL glass vial with deionized water. This dispersion was incrementally added into a 500 mL glass beaker connected to the Mastersizer until the desired obscuration percentage, ranging from 3% to 5%, was achieved. Conversely, the formulation for casting the tips of the D-MAPs did not require predispersion and was directly added to the beaker until achieving 3–5% obscuration, followed by a brief 30-s sonication. The dispersion was stirred at 2,000 rpm throughout the process. Particle sizes, specifically D10, D50, and D90, indicating the sizes at which 10%, 50%, and 90% of the particles were smaller than the given value, were recorded. All measurements were performed in triplicate.

#### 2.2.3 In Situ Insertion and Deposition of MP D-MAPs Using Porcine Skin

The insertion efficiency and *in situ* tip deposition of MP D-MAPs were studied using full-thickness excised neonatal porcine skin. The skin was excised from stillborn piglets within 24 h of birth and stored at −20 °C until needed. Skin samples were fully thawed and preequilibrated in pH 7.4 phosphate-buffered saline (PBS) for 30 min before use. MP D-MAPs were manually inserted into the skin using manual thumb pressure for 30 s. Subsequently, a cylindrical stainless-steel weight (11 mm diameter, 11.5 g mass) was placed on top to secure each D-MAP in position. To prevent moisture evaporation, the samples were wrapped in aluminum foil and placed in a 37 °C oven to simulate body temperature during the experiment. At predetermined time intervals (0, 0.5, 5, 15, 30, and 60 min), the MP D-MAPs were removed and directly examined using a Leica EZ4D stereo light microscope (Leica Microsystems, Milton Keynes, UK) to evaluate the time required for the dissolution and/or deposition of the tips *in situ*.

### 2.3 Nanocrystal-Loaded D-MAPs (NC D-MAPs)

#### 2.3.1 Fabrication of ATR NCs Using the Wet Bead-Milling Technique

ATR nanosuspensions (NSs) were developed utilizing a top-down approach and a laboratory-scale wet bead-milling technique.^41,42^ Several factors were important in defining the particle size of the nanosuspensions. These factors included the diameter of the ceramic beads used in the milling process, the glass vials volumes, the number and size of the magnet stirrers, the type and concentration of each stabilizer used in nanosuspension creation, the amount of drug added, and the milling time. In this work, three main stabilizers were investigated, those were PVA, PVP and a mixture of both in 1:1 ratio. All stabilizers were prepared in 2% w/w concentration. Table S1 (in the Supporting Information) shows how several parameters were thoroughly investigated and explored to generate various formulations. Notably, the ATR amount, stabilizer type, and stabilizer volume were purposefully changed to obtain a particle size within the range of 150–250 nm, whereas the other parameters remained constant throughout the experiment.

ATR NSs were originally produced by weighing out the required quantity of ATR into a glass vial (7 mL in volume). Following the addition of ceramic beads and magnet stirrers to the vial, the required volume of the relevant stabilizer was pipetted, as detailed in Table S1. Subsequently, each of these vials filled with the mixture was placed on an IKA stirring plate and stirred for 21 h at 1,250 rpm. Particle size (PS), polydispersity index (PDI), and zeta potential (ZP) were then measured by dispersing 20–60 μL of the resulting suspension (depending on the ATR concentration) in 3 mL of purified water.

#### 2.3.2 Lyophilization of the ATR Nanosuspensions

ATR NSs were lyophilized to concentrate the NCs within their matrices. The NSs were initially filtered via a 200-mesh nylon sieve directly into a 12 mL SpeedMixer polypropylene cup. This was a critical step in separating the NSs from the ceramic beads. Subsequently, 2 mL aliquots of deionized water were consistently used to rinse the beads and sieve. Aluminum foil was used to cover the SpeedMixer cups holding NS, and holes were created to enable air circulation throughout the freeze-drying procedure. The samples were then stored in a −80 °C freezer for at least 1 h before being transferred to a benchtop freeze drier. The pressure was kept constant at 600 mTorr throughout the cycle. Table S2 (in the Supporting Information) shows the exact parameters of the freeze–drying cycle.

#### 2.3.3 Measurement of PS, PDI and ZP

The NC PS and PDI were assessed using a Dynamic light scattering (DLS), while ZP was measured via phase analysis light scattering using a NanoBrook Omni Particle size and zeta potential analyzer (Brookhaven Instruments Corp., Holtsville, NY). Separate measurements were taken for both pre- and postlyophilization samples across all NC formulations. The samples were placed in poly(styrene) cuvette cells following their dilution in deionized water. In this study, 20–60 μL of the resultant suspension (based on ATR quantity in each sample) were dispersed in 3 mL of purified water prior to analysis. After equilibrating for 180 s at 25 °C, the PS and PDI were measured at a 90° scattering angle for 180 s. For ZP analysis, a solvent-resistant electrode was introduced into the samples, and the electrophoretic mobility of the nanoparticles (NCs) was measured at a 15° detection angle. All measurements were performed in triplicate.

#### 2.3.4 Theoretical Content of ATR in the Nanosuspensions

Throughout the lyophilization process, all water was extracted from the formulation, producing a solid, porous, sponge-like lyophilized powder. The theoretical amount of ATR in each NS was calculated by dividing the mass of the solid stabilizer by the volume of the NS. Subsequently, the percentages of polymer and ATR in the lyophilized powder were calculated and determined. Eq 1 was used to determine the predicted polymer content in milligrams, assuming a constant stabilizer concentration of 2% w/w (0.02).
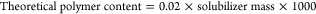
1

The percentages of ATR and polymer in each formulation were determined using eqs 2 and 3 below.
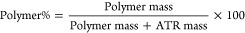
2
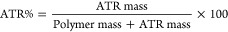
3

#### 2.3.5 The Production of Nanocrystal-Loaded D-MAPs (NC D-MAPs)

**Figure 2:**
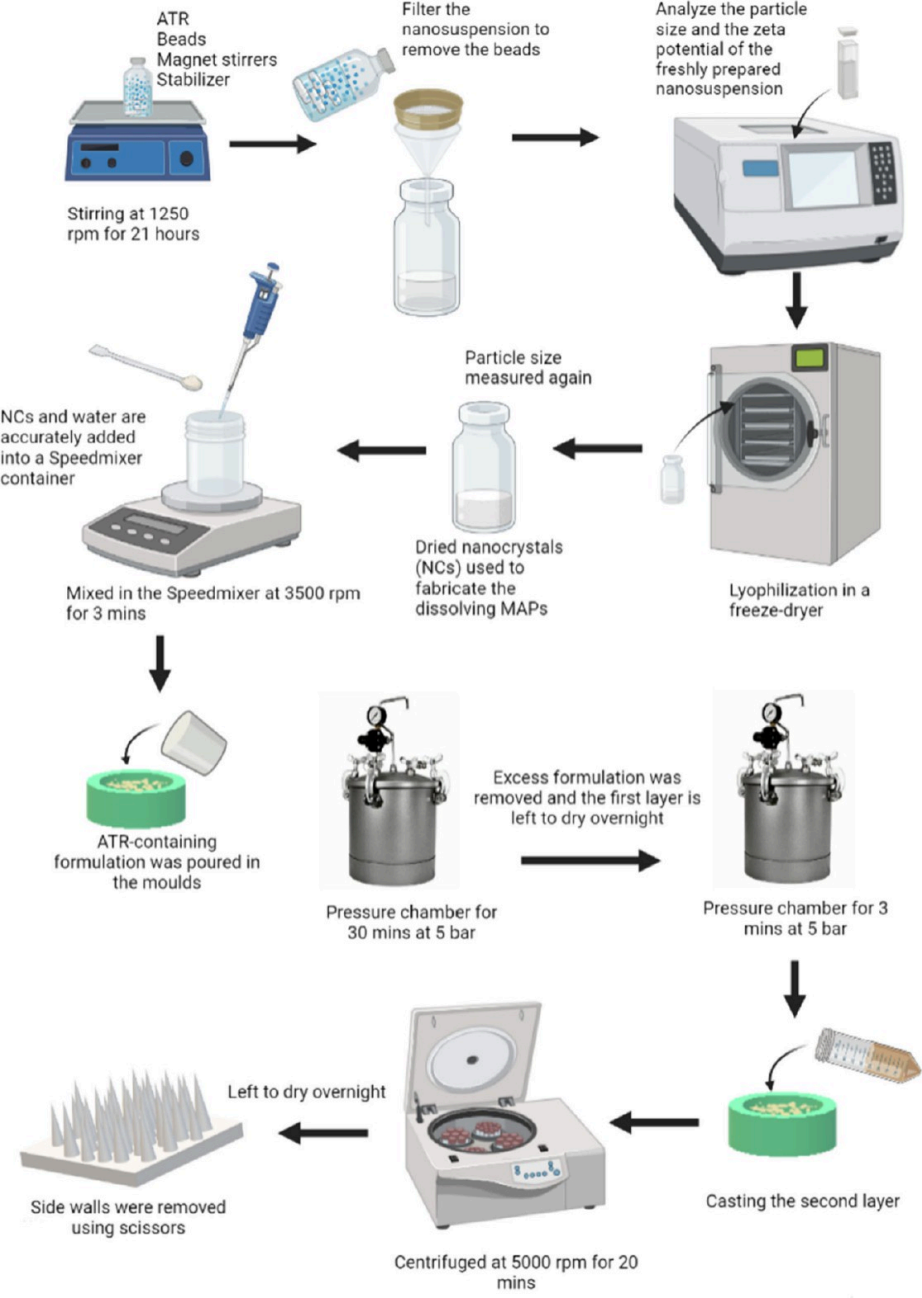
Schematic representation showing the fabrication of ATR NCs and their incorporation into D-MAPs.

For the manufacturing of NC D-MAPs, formulations with the maximum drug loading and the lowest PS were used. These formulations were cast using the previously described bilayer casting process (Section 2.2.1), with minor adjustments to the first layer preparation. The first layer in this instance was composed of the chosen NC formulation with the addition of either a 2% w/w PVA/PVP solution or pure water. The primary objective was to utilize pure water instead of the 2% w/w PVA/PVP solution to reduce polymer content in the D-MAPs and maximize drug loading. In instances where the D-MAPs produced with pure water were brittle, the 2% w/w PVA/PVP solution was employed to enhance their mechanical properties. The goal was to achieve an optimal balance between drug content and mechanical robustness of the D-MAPs. The required mass of the relevant NC formulation was precisely weighed in a 12 mL SpeedMixer cup using a four-point digital balance. Deionized water or a stabilizer was used, as indicated in Table 1, and the mixture was homogenized for 5 min using the SpeedMixer at 3,500 rpm. Positive pressure pipettes were used to cast 50 μL aliquots of the formulations into high-density molds. The molds were placed in a pressure chamber at 5 bar for 3 min, after which the surplus formulation was scrapped off their surfaces. The molds were then reintroduced into the pressure chamber for an additional 30 min. Subsequently, they were allowed to dry at room temperature for 24 h. The second layer was produced using the technique outlined in Section 2.2.1. On top of the dried first layer comprising the ATR NCs, aliquots of approximately 500 mg of the second layer were cast. After centrifuging the molds at 5,000 rpm for 20 min, they were left to dry for a further 24 h at room temperature. They were then gently demolded, and their sidewalls were cut with scissors. Figure 2 depicts the comprehensive stages in the production of the NCs and NC D-MAPs.

**Table 1:** Formulations Used in the Fabrication of the First Layer of the NC-Loaded D-MAPs

Formulation	Solvent added	NC mass (mg)	Stabilizer volume (μL)
**NC 6**	Deionized water	100	350
**NC 7**	deionized water	100	350
**NC 7**	2% PVA/PVP	100	350

#### 2.3.6 Particle Size Following the Fabrication of NC D-MAPs

The PS and PDI of the ATR NCs were assessed using DLS after the dry-optimized NC D-MAP formulation was dispersed in water. These values were then studied in comparison to the PS and PDI of the lyophilized NCs, as well as the NSs before lyophilization. All measurements were taken in triplicate.

### 2.4 Mechanical Characterization of D-MAPs

#### 2.4.1 Compression Test Using the Texture Analyzer

Using a TA-TX2 Texture Analyzer in compression mode, the mechanical characteristics of the MP D-MAPs and NC D-MAPs were investigated. A light microscope was used to examine D-MAPs from both formulations, wherein each D-MAP encompassed 600 arrays. At least 5 needles from each side of the D-MAPs were measured individually. They were then affixed using double-sided tape to a portable cylindrical probe (length 5 cm, cross-sectional area 1.5 cm^2^) stemming from the upper arm of the Texture Analyzer, with the needle side facing downward. A force of 32 N was used because it resembles the normal human thumb force.^43^ After 30 s, the D-MAPs were detached from the probe and viewed again under a light microscope. The experiment lasted 30 s before the D-MAPs were removed from the probe and inspected under a light microscope. The height reduction percentage was determined by measuring the heights of at least 5 needles from each side using eq 4, where H0 is the MN height before the test and H1 is the MN height after the test.
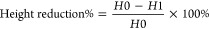
4

#### 2.4.2 In Vitro Skin Insertion Using an Artificial Skin Model

The efficiency of the *in vitro* insertion of both MP D-MAPs and NC D-MAPs was assessed using an 8-layer Parafilm M stack. This stack was constructed and placed on the TA-TX2 Texture Analyzer base plate. As previously stated, Parafilm M was used as a verified *in vitro* skin model.^43^ The D-MAPs were affixed to a movable cylindrical probe with the needles facing downward. The probe was then pressed into a stack of Parafilm M with a force of 32 N for 30 s. After removal, the Parafilm M layers were separated, and the number of holes created was counted using a light microscope. The insertion percentage was calculated using eq 5.
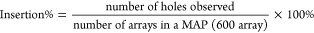
5

#### 2.4.3 Ex Vivo Skin Insertion Efficiency via Optical Coherence Tomography

To assess the insertion efficiency of both D-MAP formulations into excised neonatal porcine skin *ex vivo*, Optical Coherence Tomography (OCT) microscope (Michelson Diagnostics Ltd., Kent, UK) was used. Within 24 h of birth, skin samples were collected from stillborn piglets. The full-thickness skin was gently shaved with a razor and preserved in plastic Petri dishes at −20 °C until required. Before the experiment, the skin was completely thawed in PBS (pH 7.4) for approximately 30 min. D-MAPs from both formulations were inserted manually, using firm thumb pressure for 30 s, and then visualized using OCT.

### 2.5 DSC, TGA, ATR-FTIR, and PXRD Studies

Analyses were performed on samples containing the crude ATR powder, MP-loaded formulation, NC-loaded formulations, PVA and PVP powders, and their physical mixtures. Thermogravimetric analysis (TGA) and differential scanning calorimetry (DSC) were performed using TGA Q50 and DSC Q100 (TA Instruments, Elstree, Hertfordshire, UK). Furthermore, attenuated total reflectance-Fourier transform infrared (ATR-FTIR) spectroscopy experiments were conducted utilizing an Accutrac FT/IR-4100TM spectrometer (PerkinElmer, USA). PXRD studies were performed using a MiniFlex II Desktop Powder X-ray Diffractometer (Rigaku Corporation, Kent, England).

### 2.6 Actual ATR Content in D-MAPs

The actual drug content of the D-MAPs formulation was studied. The sidewalls of the four D-MAPs were carefully removed, and the MAPs were dissolved in glass vials encompassing a solution of 1% w/v SLS in PBS (pH 7.4). This solvent was chosen based on the preliminary saturation solubility study findings reported previously.^1^ The samples were stirred at 1,500 rpm until the D-MAPs were completely dissolved. Following a 2 h sonication, the vials were diluted in acetonitrile and methanol to precipitate the polymers, and then they were diluted again in 1% w/v SLS in PBS (pH 7.4). The samples were centrifuged at 10,000 rpm for 10 min, then filtered through 0.2 m Minisart syringe filter. RP-HPLC analysis was then performed on the samples.

### 2.7 Ex Vivo Skin Deposition Studies

#### 2.7.1 In Situ Insertion and Deposition of MP-Loaded D-MAPs

This study examined the insertion efficiency and tip deposition of MP D-MAPs in full-thickness excised neonatal porcine skin. The porcine skin was excised from stillborn piglets within 24 h of birth and preserved at −20 °C until use. Prior to the experiment, the skin samples were preequilibrated in PBS (pH 7.4) for 30 min. The MP D-MAPs were manually implanted into the skin for 30 s using thumb pressure, and a cylindrical stainless-steel weight (11 mm in diameter, 11.5 g mass) was placed on top to secure the D-MAP in place. The samples were coated in aluminum foil to prevent moisture evaporation while being stored in a 37 °C oven to simulate body temperature conditions during the experiment.

#### 2.7.2 ATR Extraction Method from Skin Samples

The extraction efficiency of ATR from skin samples was evaluated through the development and validation of an extraction procedure prior to the start of *ex vivo* skin deposition studies. To facilitate injection, the aqueous ATR formulation outlined in Section 2.2.1 was produced and diluted 1:1 with H_2_O. The full thickness excised porcine skin samples were shaved and balanced in PBS (pH 7.4) before being cut into smaller pieces. Each piece was weighed individually, and the ATR formulation was cautiously injected with a syringe between the epidermis and dermis layers. After injection, the samples were reweighed to determine the mass of the injected formulation and the corresponding amount of ATR in each skin sample.

Skin samples were then put on weighing boats over tissue papers soaked in PBS (pH 7.4) and covered with aluminum foil to prevent drying. They were incubated in a 37 °C oven for 24 h. After incubation, the skin samples were cut into smaller pieces and placed in 2 mL microtubes. Each tube was filled with 0.5 mL of deionized water and homogenized for 15 min at 50 Hz using a Tissue Lyser LT. Thereafter, 1 mL of methanol was added into each tube, and the samples were homogenized again for 15 min at 50 Hz.

Each mixture was then sonicated for 2 h in a 15 mL Falcon tube containing 3.5 mL of methanol. Thereafter, the samples were thoroughly vortexed, diluted at 10 times using 1% w/v SLS in PBS (pH 7.4), and centrifuged at 14,800 rpm for 30 min. The diluted samples were filtered using 0.2 μm Minisart syringe filters before analysis via RP-HPLC.

#### 2.7.3 Ex Vivo Skin Deposition Using Franz Cells

Modified Franz diffusion cells were employed to perform the *ex vivo* skin deposition studies. Full-thickness skin was excised within 24 h of birth and preequilibrated in PBS at pH 7.4 for 30 min on the experimental day. The shaved skin was attached to the donor compartment of the Franz diffusion cells using cyanoacrylate glue, to ensure and maintain adhesion during and after MAPs insertion. D-MAPs from the two main formulations were manually inserted into the skin by exerting firm thumb pressure for 30 s. A few dental wax pieces wrapped in aluminum foil was used as a support during the insertion process, to ensure that the MNs tips remain intact following insertion. A stainless-steel cylinder (11 mm diameter, 11.5 g mass) was placed on the upper surface of the D-MAPs to hold them in place. The receiver chamber was filled with 12 mL of 1% w/v SLS in PBS (pH 7.4). To avert solvent evaporation, the assembled setup was wrapped with Parafilm M. At 24 h, the Franz cells were dismantled, and ATR was extracted from the skin (as indicated in Section 2.8.2). ATR delivered throughout 24 h was quantified by analyzing the samples obtained from the receiver compartment. The total amount of ATR delivered to the skin and receiver compartments was determined.

To ensure specific drug quantification from the skin, excess formulation remaining on the skin surface at 24 h had to be removed. This was accomplished by adding 25 μL aliquots of PBS to the skin and lightly tapping it with tissue paper. A 4-h time point was also investigated for MP D-MAPs. At this point, all Franz cells were disassembled, and samples from the receiver compartment and skin were collected and examined to compare the amount of intradermal ATR deposited at 4 and 24 h, with the goal of examining the impact of shorter wear duration on drug deposition.

### 2.8 In Vivo Depot Delivery of ATR from Sprague–Dawley Rats

#### 2.8.1 In Vivo Study Design and Execution

The Biological Services Unit Committee at Queen’s University Belfast approved the animal studies under Project License PPL 2904 and Personal Licenses PIL 1892, PIL 1747, PIL 2056, and PIL 2202. With a dedication to applying the 3Rs principle (replacement, reduction, and refinement), all in vivo experiments were carried out in accordance with the regulations of the European Convention for the Protection of Vertebrate Animals used for Experimental and Other Scientific Purposes and the Federation of European Laboratory Animal Science Associations.

This study serves as an additional investigation to follow-up a larger study reported by Naser et al. in 2023.^1^ In that study, the first successful utilization of hydrogel-forming MAPs for the long-acting delivery of ATR was reported and compared to that in an oral control cohort. To minimize the number of animals used, in compliance with the 3Rs principle in animal experimentation, and to prioritize humane practices in conducting animal work, the control group was not replicated in this study. Herein, female Sprague–Dawley rats (total n = 6), aged 8–10 weeks with a mean weight of 263.8 ± 29.8 g, were acclimatized for 7 days in separate cages within the animal facility prior to the experiment. Each rat received 4 MP D-MAPs containing ATR, totaling a dose equivalent to 20 mg/rat. In our previous study, the oral control group received an ATR suspension at a dose of 40 mg/kg (equivalent to 10 mg/rat) via oral gavage. This dose was chosen based on the literature.^44^

Dorsal hair was removed from the rats prior to the experiment to reduce interference from hair during the MAPs application. Rats were initially sedated by administering a gaseous anesthetic (2–4% v/v isoflurane in oxygen), and then the bulk hair was shaved using electric clippers. The remaining hair was removed using a depilatory hair removal cream. Afterward, the rats were left for 24 h to allow the recovery of the skin and to ensure the full restoration of skin barrier function before the MAPs were inserted.^15,45^

**Figure 3:**
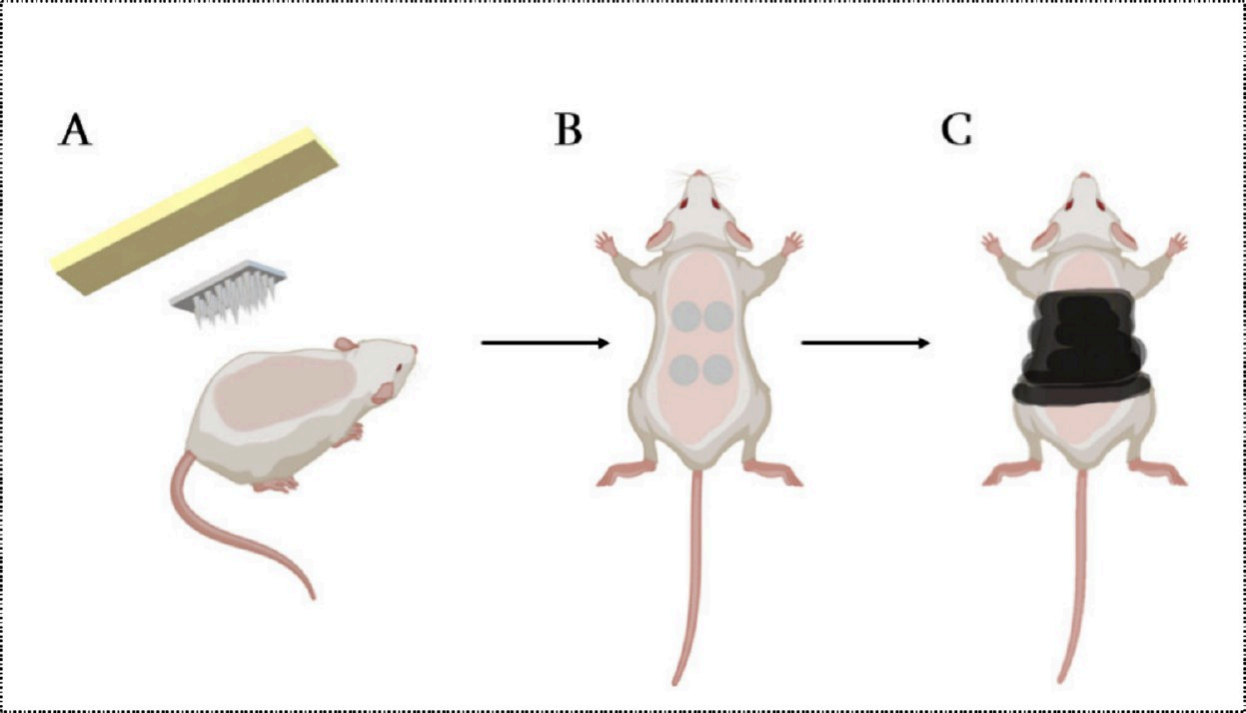
(A–C) A schematic representation illustrating the application and assembly of D-MAPs for the in vivo study. Modified with permission from ref (1). Copyright 2023 The Authors. Published by Elsevier B.V.

The rats were sedated again on the following day with the same anesthetic gas, and 4 MP D-MAPs were inserted into their shaved dorsal area using firm thumb pressure for 30 s. Figure 3A and B show Microfoam surgical adhesive tape placed on top of the MAPs. Tegaderm film was then applied on top of the MP D-MAPs, and kinesiology tape was then wrapped around the rats’ backs for 24 h to firmly hold the MP D-MAPs in place (Figure 3C). Each rat was individually housed for 24 h following the MP D-MAP application to guarantee that the D-MAPs remained in place. Blood samples were collected into preheparinized 1.5 mL microtubes via tail vein bleeds throughout a 14-day period at predefined time intervals of 1, 2, 4, 6, 24, 48, 72, 96, 168, 240, and 336 h.

#### 2.8.2 Sample Preparation and ATR Extraction from Plasma

To separate plasma from other blood components, samples acquired from rats were immediately centrifuged at 2,200 × g for 10 min at 4 °C. The supernatant (plasma) from each sample was carefully transferred to its corresponding labeled microtube and stored at −20 °C until further analysis.

To extract ATR from plasma samples, 300 μL of methanol was added to 100 μL aliquots of plasma in each microtube. The mixtures were vortexed for 25 min at 1,500 rpm. Thereafter, the samples were centrifuged at 14,000 × g for 10 min at 4 °C. The supernatants were subsequently transferred into glass inserts in HPLC vials for subsequent LC MS/MS analysis.

#### 2.8.3 Pharmacokinetic Analysis of ATR and Relative Bioavailability

Noncompartmental pharmacokinetic (PK) analysis of ATR plasma concentrations was performed using PK Solver, a Microsoft Excel add-in program (Microsoft Corporation, Redmond, WA, USA). Drug concentrations (ng/mL) were plotted against time (days). The raw data was used for the direct extraction of maximum plasma concentration (C max) and the time to reach this maximum (T max). Additional key parameters calculated included the area under the concentration–time curve from zero to the last experimental time point (AUC 0-t), the area under the concentration–time curve extrapolated to infinity (AUC 0-inf_obs), and the mean residence time (MRT).

Relative bioavailability is defined as the amount of drug reaching the systemic circulation from one formulation compared to another nonintravenous (IV) formulation, such as an oral solution.^46^ Using eq 6 below, the transdermal bioavailability of ATR was determined.
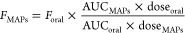
6

### 2.9 Pharmaceutical Analysis

The quantification of ATR was performed as previously described by Naser et al.^1^ The details of the method are summarized in Table S3 in the Supporting Information.

### 2.10 Statistical Analysis

The statistical analysis was performed using GraphPad Prism version 9.0 (GraphPad Software, San Diego, California, USA). The Shapiro–Wilk test was used to assess the normality of the collected data. For normally distributed data (parametric data), an unpaired *t* test was used to compare two groups, while one-way ANOVA and two-way ANOVA with Tukey’s post hoc test were used to examine differences across groups (≥3). All data are presented as means ± standard deviation. Statistical significance was indicated by a *p* < 0.05.

## 3 Results and Discussion

### 3.1 MP-Loaded D-MAPs (MP D-MAPs)

#### 3.1.1 Fabrication of ATR Microparticle-Loaded D-MAPs

**Figure 4:**
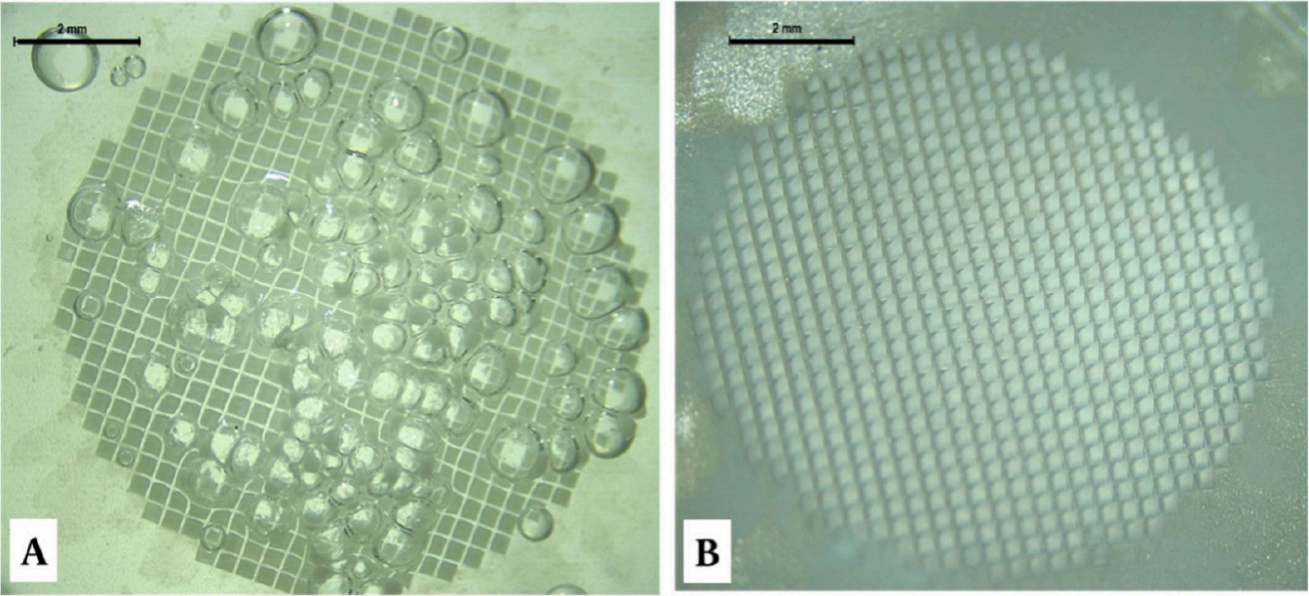
ATR MP-loaded D-MAPs after casting the second layer. (A) Four hours after the first layer and (B) 24 h after casting the first layer (overnight). The scale bar represents 2 mm in length.

After the MP D-MAPs had dried completely, they were carefully demolded, and their sidewalls were gently removed using scissors. Following that, the MP D-MAPs were examined with a Leica EZ4D light microscope, where the images are presented in Figure 4. When dried for only 4 h, bubbles were observed to form between the two casted layers as shown in Figure 4A. This finding can be essentially ascribed to the hydrophobic nature of ATR. The high ATR content in the first layer (approximately 60% w/w) may have increased the hydrophobicity of this layer. Hydrophobic surfaces are distinguished by their water inertness, which means that they are incapable of forming hydrogen bonds or electrostatic interactions with water molecules. This could result in long-chain force hydrophobic interaction.^47^ Therefore, repulsive electrostatic forces between the hydrophilic second layer and the hydrophobic ATR might have occurred when the second layer was cast before the complete drying of the first layer,^47^ resulting in the production of bubbles on the surface of the D-MAPs.

The batch that was allowed to cure for 24 h, on the other hand, was fully formed, and ATR was uniformly dispersed after casting and drying, with no bubbles visible on their surfaces (Figure 4 B). This can be attributed to the thorough drying of the formulation, which reduced the interactions between the hydrophobic ATR and the hydrophilic backing layer.

#### 3.1.2 Particle Size of the ATR Powder and D-MAPs Formulation

In this study, MP manufacturing was carried out in a straightforward process using only SpeedMixer. Here, the high level of agitation applied in the Speedmixer causes particle aggregates that are electrostatically attached to separate into smaller clusters. This phenomenon is facilitated by the presence of PVP and PVA, which quickly surround each separate cluster, stabilizing them in their deaggregated form, thereby reducing the overall mean particle size. In other words, this process forms a deaggregated drug suspension rather than milling the particles.^48^ To create these MPs, no milling or solvent evaporation procedures were required. Alternatively, the addition and mixing of ATR, PVA and PVP in SpeedMixer was the exact step used to produce the MPs. This approach makes the production process simple, cost-effective, and easily scalable. The results are detailed in Table 2 below. Notably, 90% of the ATR particles in the MP formulation were less than 41 μm in size, with 50% being less than 7 μm. This size was approximately half of that of the crude ATR powder. Even though ATR consists of small particles, mixing the powder with polymers in the SpeedMixer was critical for reducing the particle size by half. Moreover, the ATR particles distribution was more uniform in the presence of MP than the original crude powder of ATR. Furthermore, compared to that of the original crude ATR powder, the particle distribution of the MPs was more uniform. The polymers used in MP and D-MAP preparation (PVA and PVP) are known to be safe, biocompatible, and widely used, with no previous safety issues recorded,^49^ which could indicate their successful implementation in D-MAP preparation.^24,26,30,31,40^

**Table 2:** Mean Diameter of Percentage Undersize Volume Fraction of the ATR MP Formulation and Pure ATR Powder^a^

Sample	D_10_ (μm)	D_50_ (μm)	D_90_ (μm)
MP	3.00 ± 0.09	6.81 ± 0.22	40.90 ± 1.56
ATR powder	4.41 ± 0.17	21.33 ± 3.25	80.6 ± 2.43

a Means ± SD, *n* = 3.

#### 3.1.3 In Situ Insertion and Dissolution of MP-Loaded D-MAPs into Full-Thickness Porcine Skin

**Figure 5:**
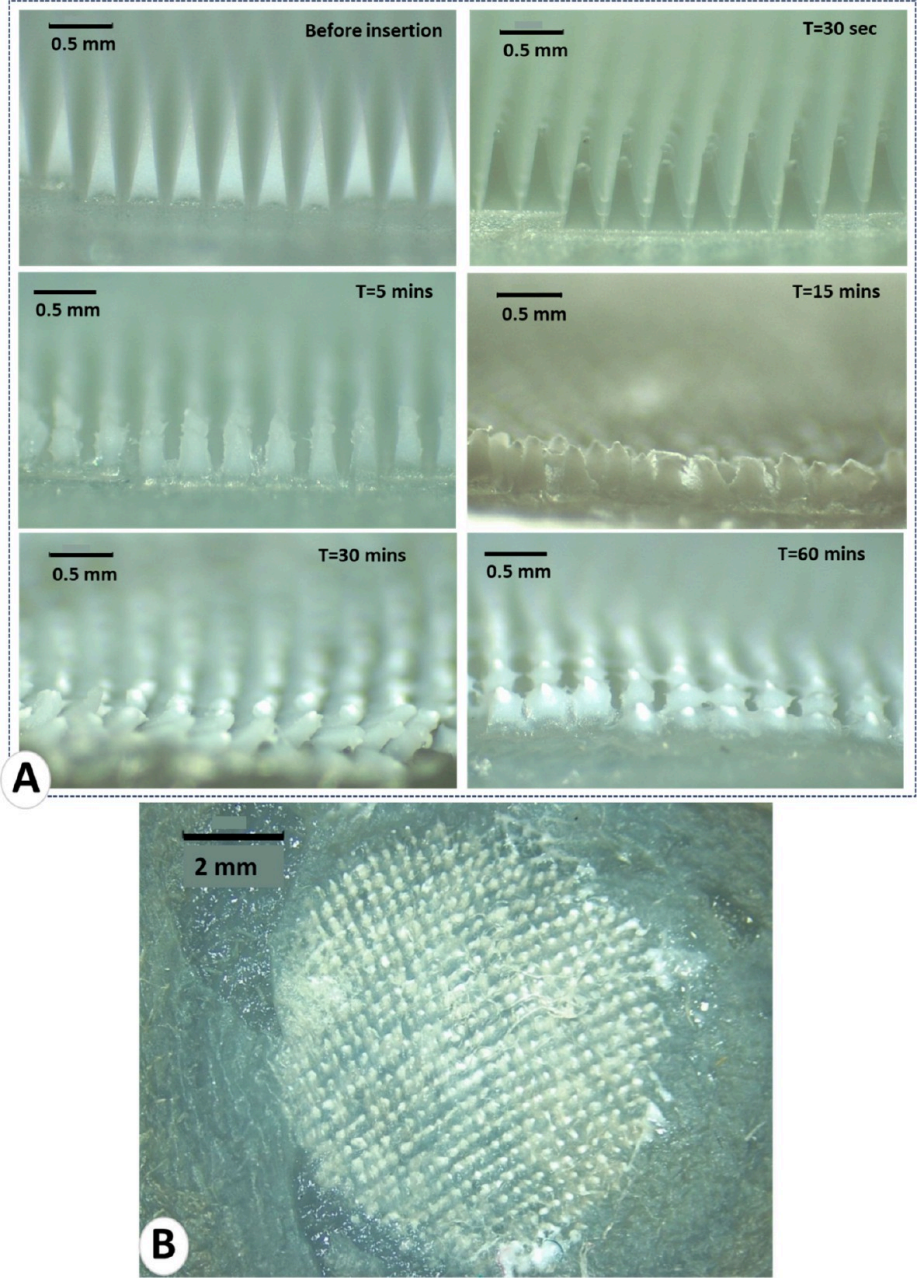
(A) Digital images depict micrographs of in situ insertion and dissolution of the tips of D-MAPs containing ATR microparticles at specified time points: 0, 0.5, 5, 15, 30, and 60 min following insertion into and removal from excised neonatal porcine skin. Each image includes a scale bar representing 0.5 mm. (B) An image of the ATR MP D-MAPs patch after 60 min of insertion into excised neonatal porcine skin, showing complete deposition of the tips into the skin. The black scale bar represents a length of 2 mm.

Deposition and dissolution studies were performed *in situ* to predict the time required for the complete deposition of needles from MP D-MAPs following their implantation into the skin. D-MAPs were examined using a light microscope before insertion into the skin (at *t* = 0) and then at 30 s, 5, 15, 30, and 60 min, as shown in Figure 5A. Encouragingly, the D-MAPs commenced dissolution within 5 min of being inserted into the skin. Within 60 min, the tips had nearly completely dissolved, subsequently detaching from the baseplate and embedding effectively in the skin, as shown in Figure 5B.

### 3.2 Nanocrystal-Loaded D-MAPs (NC D-MAPs)

#### 3.2.1 Fabrication of ATR NCs Using the Wet Bead Milling Technique

Initially, ATR NCs with particle sizes ranging from 150 to 250 nm were produced using the wet bead-milling technique to increase the drug loading in the D-MAPs. This was aimed because the benefits of reducing particle size on drug dissolution rate become more evident when the mean particle size is below 1 μm.^48^ This approach, noted for its industrial scalability, is regarded as a universal strategy in the fabrication of NCs, notably for enhancing the solubility of hydrophobic drugs.^50^Figure S1 of the Supporting Information shows that changing the stabilizer type or volume did not result in a significant change in particle size (*p* > 0.05). However, compared to the 100 mg loading, the addition of 300 mg of ATR to the NSs resulted in a significantly larger particle size (*p* < 0.037). Nonetheless, no statistically significant difference was found among the other formulations. Regarding PDI, loading higher amounts of the drug (300 and 400 mg) resulted in significantly lower (*p* < 0.05) values than loading lower amounts (100 and 200 mg). As for ZP, all formulations had positive charges, with no significant difference noted across varying parameters.

#### 3.2.2 Lyophilization of ATR Nanosuspensions

To stabilize the NS and serve as cryoprotectants throughout the lyophilization process, PVA 9–10 kDa, PVP 58 kDa, and a mixture of both polymers were used. The effects of ATR loading, stabilizer volume, and type on PS, PDI, and ZP in lyophilized NCs were investigated, and the findings are reported in Figure S2 of the Supporting Information. Interestingly, no significant difference (*p* > 0.05) in any parameter was noted before and after lyophilization of the NS. This shows that PVA and PVP, employed as stabilizers in the NCs manufacturing process, served efficiently as cryoprotectants. These compounds were critical for shielding the NCs during the lyophilization process, as evidenced by the results shown in Figure S1 and S2 in the Supporting Information. Furthermore, these polymers displayed the ability to stabilize NCs even during the lyophilization process, resulting in the creation of small nanocrystals with particle sizes ranging from 180 to 230 nm.

#### 3.2.3 Theoretical ATR Content in the Nanosuspensions

The results of determining the theoretical ATR-to-polymer proportion in the dry lyophilized NC formulation are summarized in Table 3. All formulations had varying amounts of ATR, ranging from 45.5% to 80% by weight. PVA, PVP, and a 1:1 w/w combination of the two were investigated considering their excellent biocompatibility and safety profiles, in addition to their ability to produce D-MAPs with robust mechanical properties.^24^

**Table 3:** Summary of the Theoretical ATR-to-Polymer Ratios of the Lyophilized NCs

NC	ATR (mg)	Polymer type	Polymer quantity (mg)	ATR:polymer (%)
**NC 1**	100	PVA	120	45.5%:54.5%
**NC 2**	200	PVA	120	62.5%:37.5%
**NC 3**	300	PVA	120	71.4%:28.6%
**NC 4**	100	PVA/PVP	120	45.5%:54.5%
**NC 5**	200	PVA/PVP	120	62.5%:37.5%
**NC 6**	300	PVA/PVP	120	71.4%:29.6%
**NC 7**	400	PVA/PVP	100	80%:20%
**NC 8**	400	PVA	100	80%:20%
**NC 9**	100	PVP	120	45.5%:54.5%
**NC 10**	200	PVP	120	62.5%:37.5%

Given the preceding results, this study delved more deeply into NC formulations containing 300 mg and 400 mg of ATR to assess drug loading to achieve the objective of enhancing the drug loading of the formulation in D-MAPs. Considering that the type of stabilizer did not have a significant impact on the PS reduction (as indicated in Figure S1 and S2 in the Supporting Information), a combination of PVA and PVP was selected for two key reasons. First, the polymer mixture can impart more favorable mechanical characteristics on the D-MAPs compared to each polymer separately, as previously demonstrated.^24^ Second, as PVA and PVP mixture was also used in the preparation of MP D-MAPs, the decision was made to maintain consistency among the formulations. Therefore, NC 6 and NC 7 were selected for further investigations. Additionally, PVA and PVP exhibit an excellent biocompatibility profile, are safe, cost-effective,^32,40,49,51^ and provide sufficient mechanical strength to be used in the D-MAPs matrices.^49,52^ This simplified the D-MAPs fabrication process and eliminated the requirement of adding more polymers to the formulation to provide enough mechanical support.

#### 3.2.4 Fabrication of NC-Loaded D-MAPs

D-MAPs produced from NC 6 and NC 7 formulations were examined under a light microscope and the results are described in Table 4. F1 and F3 both resulted in fully formed D-MAPs, with ATR NCs homogeneously dispersed within the tips. Nevertheless, bubble formation was observed on the surfaces of F2, and ATR was not evenly dispersed within their tips, resulting in incomplete MN formation. Additionally, F2 tips were brittle and susceptible to breaking during the demolding process. These findings can be mainly attributed to the lower polymer content in F2 compared to F1 and F3, resulting in reduced mechanical robustness. This was resolved when water was substituted with a 2% PVA/PVP solution in F3, confirming the previous hypothesis of low polymer content. Therefore, F2 was excluded at this stage, and only F1 and F3 were chosen for subsequent studies.

**Table 4:**
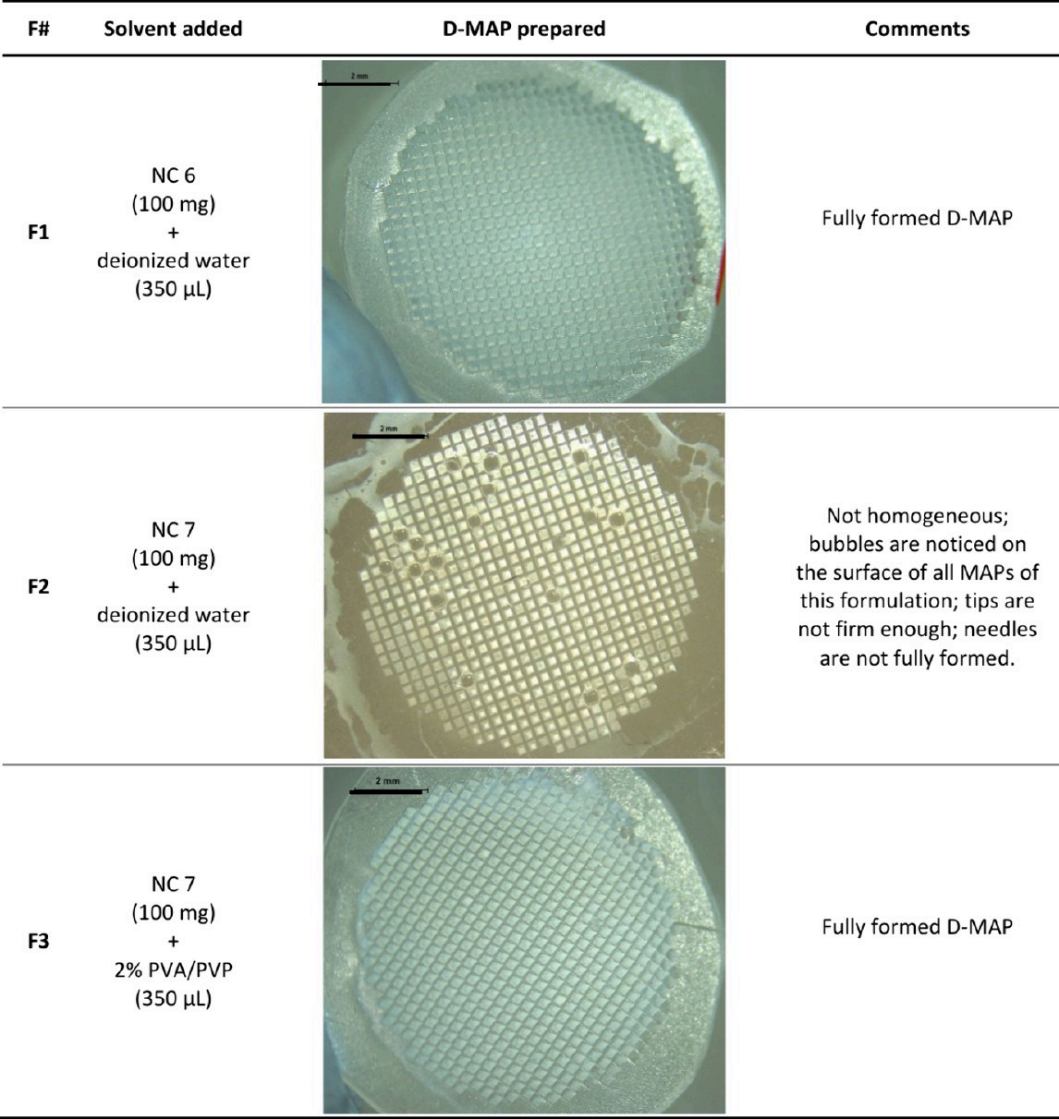
NC Formulations Used in the Production of NC D-MAPs^a^

a The black scale bar in all images represents a length of 2 mm.

**Figure 6:**
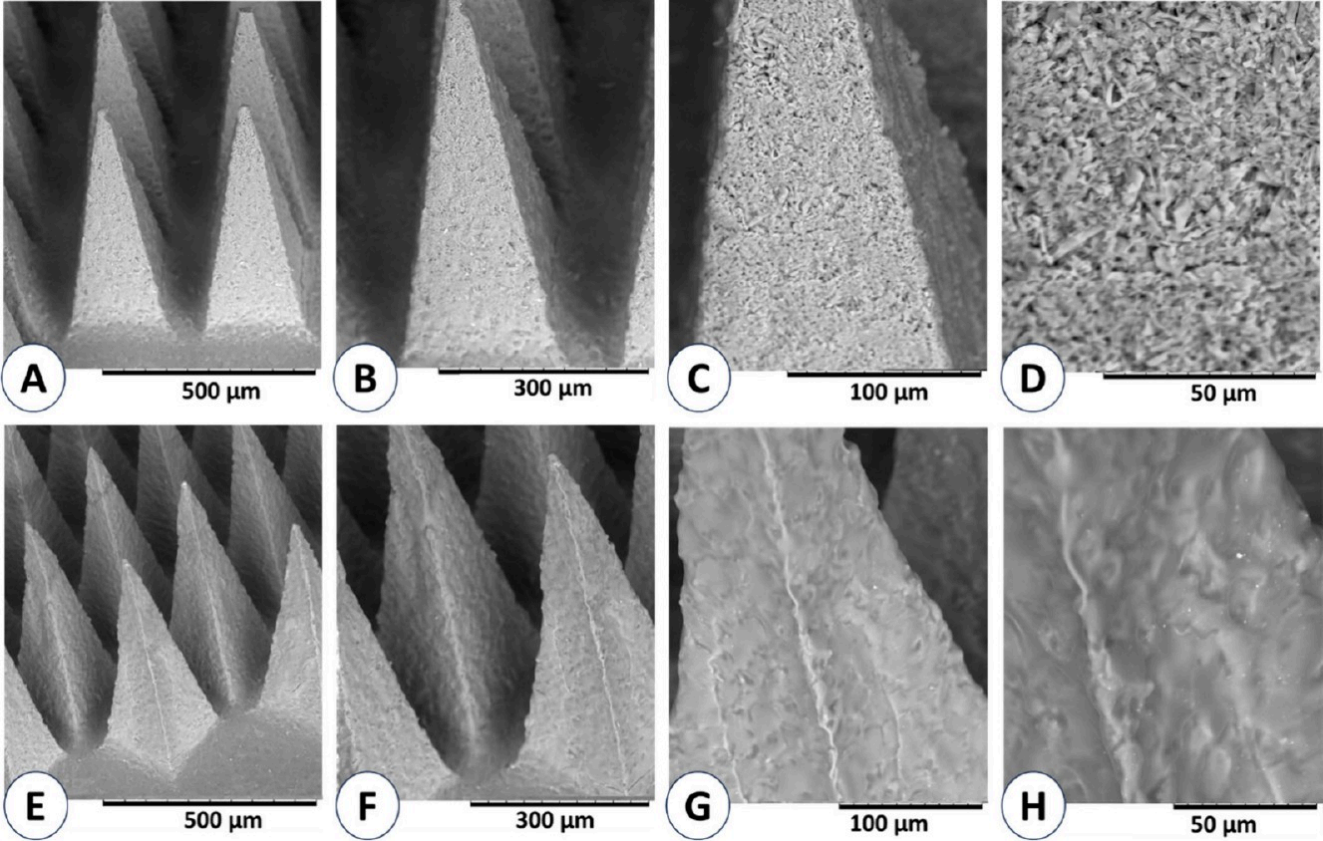
(A–D) Show SEM images of MP D-MAPs using different magnifications. (E–H) Present SEM images of NC D-MAPs.

Scanning electron microscope (SEM) images showing the morphology of fully formed MP D-MAPs and the NC D-MAPs of choice were captured and are presented in Figure 6 below.

#### 3.2.5 Particle Size Following the Fabrication of NC D-MAPs

DLS was used to determine the PS and PDI of the NSs (NC 6 and NC 7) before lyophilization, as well as the lyophilized NC powder and the dry rationalized NC D-MAP formulation after dispersion in deionized water. The results are summarized in Table 5. After lyophilization, the PS of NC 6 increased from 185.74 ± 2.54 nm to 195.45 ± 4.14 nm (*p* = 0.0046), and after casting the D-MAPs, it further increased to 220.18 ± 2.37 nm. Similar trends were observed for NC 7, where the PS increased slightly after lyophilization, from 189.98 ± 0.5 nm to 200.34 ± 1.15 nm (*p* = 0.0027), and after casting the D-MAPs, from 200.34 ± 1.15 nm to 233.10 ± 1.42 nm (*p* < 0.0001). Despite the size changes during lyophilization and casting, the PS of the ATR NCs remained below 250 nm in both cases following casting the D-MAPs. Additionally, NC 6 possessed a significantly lower PS than NC 7 after casting to prepare the MAPs (*p* = 0.0004).

**Table 5:** PS, PDI, and ZP of the Two-Lead Nanocrystal Formulation Pre- and Postloading into D-MAPs^a^

Formulation code and status		PS	PDI
NC 6	Before lyophilization	185.74 ± 2.54	0.103 ± 0.044
	After lyophilization	195.45 ± 4.14	0.119 ± 0.023
	After casting MAPs	220.18 ± 2.37	0.115 ± 0.011
NC 7	Before lyophilization	189.98 ± 0.5	0.099 ± 0.019
	After lyophilization	200.34 ± 1.15	0.099 ± 0.035
	After casting MAPs	233.10 ± 1.42	0.087 ± 0.014

a Means ± SDs, *n* = 3.

### 3.3 Mechanical Characterization of D-MAPs

#### 3.3.1 Compression Test Using the Texture Analyzer

**Figure 7:**
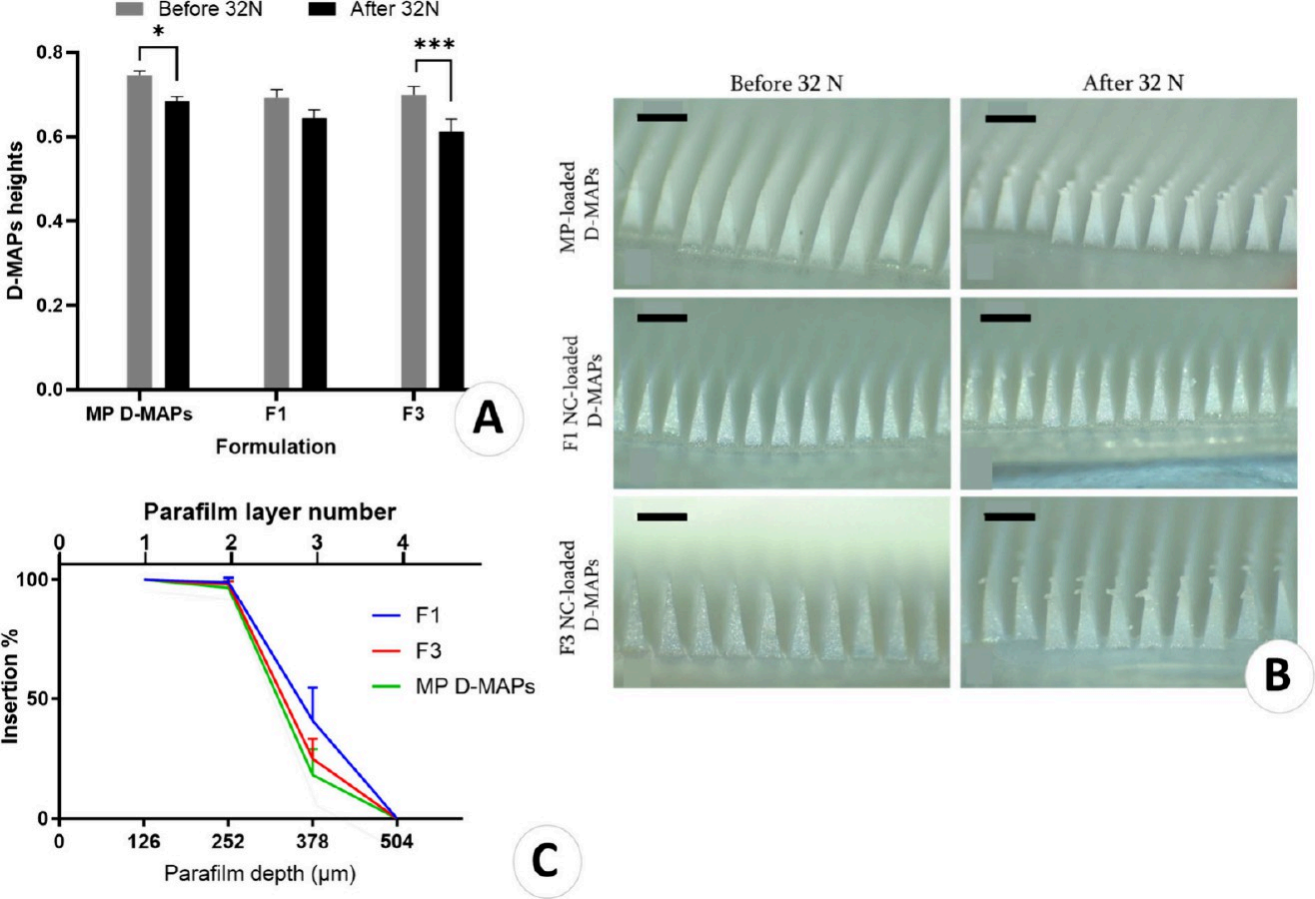
(A) Heights of D-MAPs before and after applying 32 N force for 30 s against a stainless-steel block. (Means + SDs, *n* = 3) (*p = 0.02, **p = 0.0007). (B) MP D-MAPs, F1 and F2 final D-MAPs before and after the application of 32 N of force during the compression test, demonstrating bending in their tips. Scale bars represent 0.5 mm length. (C) Percentage of holes created in each Parafilm M layer and approximate insertion depth of each formulation of the D-MAPs. (Means ± SDs, *n* = 3).

The mechanical robustness of the final three candidate D-MAPs formulations—MP D-MAPs, F1, and F3—was assessed by determining the height reduction percentage. This parameter serves as an indicator of their ability to withstand pressure when applied to the skin and avoid breakage.^53^ The results, depicted in Figure 7A, showed height reduction percentages of 8.27%, 6.96%, and 12.51% for MP D-MAPs, F1, and F3, respectively. A significant difference in the heights of the D-MAPs was observed between the MP-loaded formulation and F3 (*p* < 0.05), prior to and post compression. In contrast, F1 did not exhibit a significant reduction in the height of the arrays.

Importantly, none of the D-MAPs formulations broke during the compression test; however, they were bending, as seen in Figure 7B. This suggests that all the D-MAPs formulations could withstand the pressure, emphasizing their safety and resilience during the application process.

#### 3.3.2 In Vitro Skin Insertion Using an Artificial Skin Model

Figure 7C depicts the insertion percentages of D-MAPs through the Parafilm M stack, showing that all D-MAPs formulations successfully punctured the first two layers of Parafilm M, attaining an insertion depth of >252 μm. Nevertheless, there were differences in the ability of the formulations in penetrating the third layer, with depths ranging from 378 to 507 μm. Particularly, there was no significant difference in their insertion efficiency into the third Parafilm M layer (*p* > 0.05). This corresponds to approximately 50–70% of the needle heights, which is consistent with previous findings reported in the literature.^23,28,54^ The epidermis thickness falls within the 100–150 μm range, whereas the thickness of the dermis is within 0.5 to 5 mm and is likely to reach the body.^55−57^ Given these parameters, it is reasonable to assume that all D-MAPs formulations can penetrate the dermal layers of the skin. This approach is especially beneficial because the dermis is a noninnervated region, making D-MAP technology suitable for painless delivery of molecules across the skin.^58^

Considering the previous results, two formulations were chosen for further investigation: MP D-MAPs and F1 of the NC D-MAPs. In comparison to F3, both formulations demonstrated lower height reduction percentages. They also had superior insertion efficiency into the Parafilm M stack, although the difference was not statistically significant. Notably, compared to F3, F1 had a significantly smaller nanocrystal (NC) size after casting the D-MAPs, as previously shown in Table 5.

#### 3.3.3 Ex Vivo Skin Insertion Efficiency

**Figure 8:**
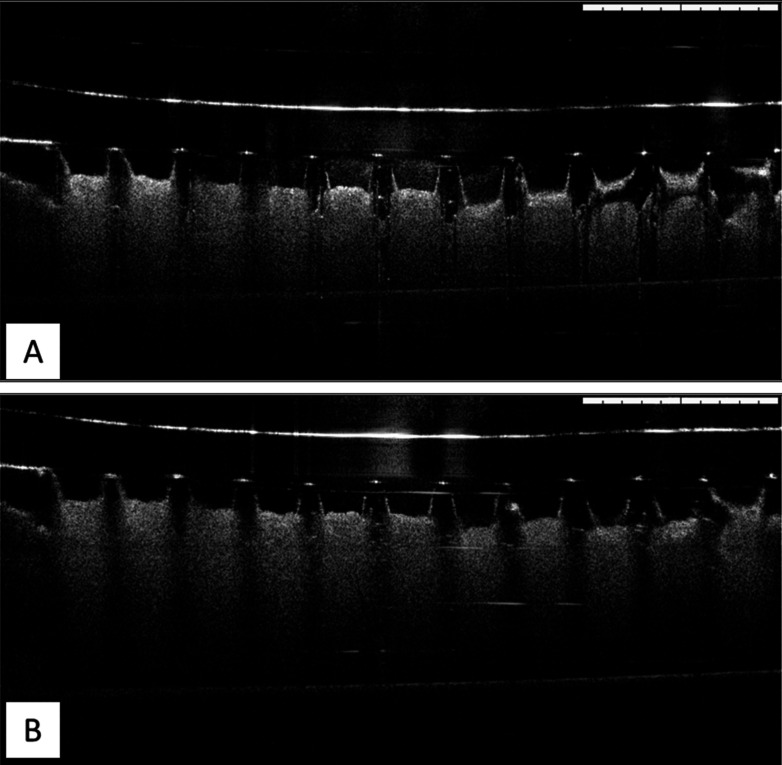
Representative OCT images following the ex vivo insertion of the (A) MP D-MAPs formulation and (B) F1 D-MAPs formulation (containing NC 6) into excised full-thickness neonatal porcine skin. The white scale bar represents a 1 mm length.

OCT was used to assess the *ex vivo* insertion efficiency of the D-MAPs into excised full-thickness neonatal porcine skin. As shown in Figure 8, MP-loaded D-MAPs possessed an insertion depth of 644.13 ± 17.10 μm (Figure 8A), and F1 (NC 6 D-MAPs) had an insertion depth of 635.98 ± 19.98 μm (Figure 8B). Notably, there was no statistically significant difference in insertion depth between the two lead formulations (*p* > 0.05). The needle heights inserted in this study were comparable to heights obtained previously in other studies.^43,59^

### 3.4 DSC, TGA, ATR-FTIR, and PXRD Studies

**Figure 9:**
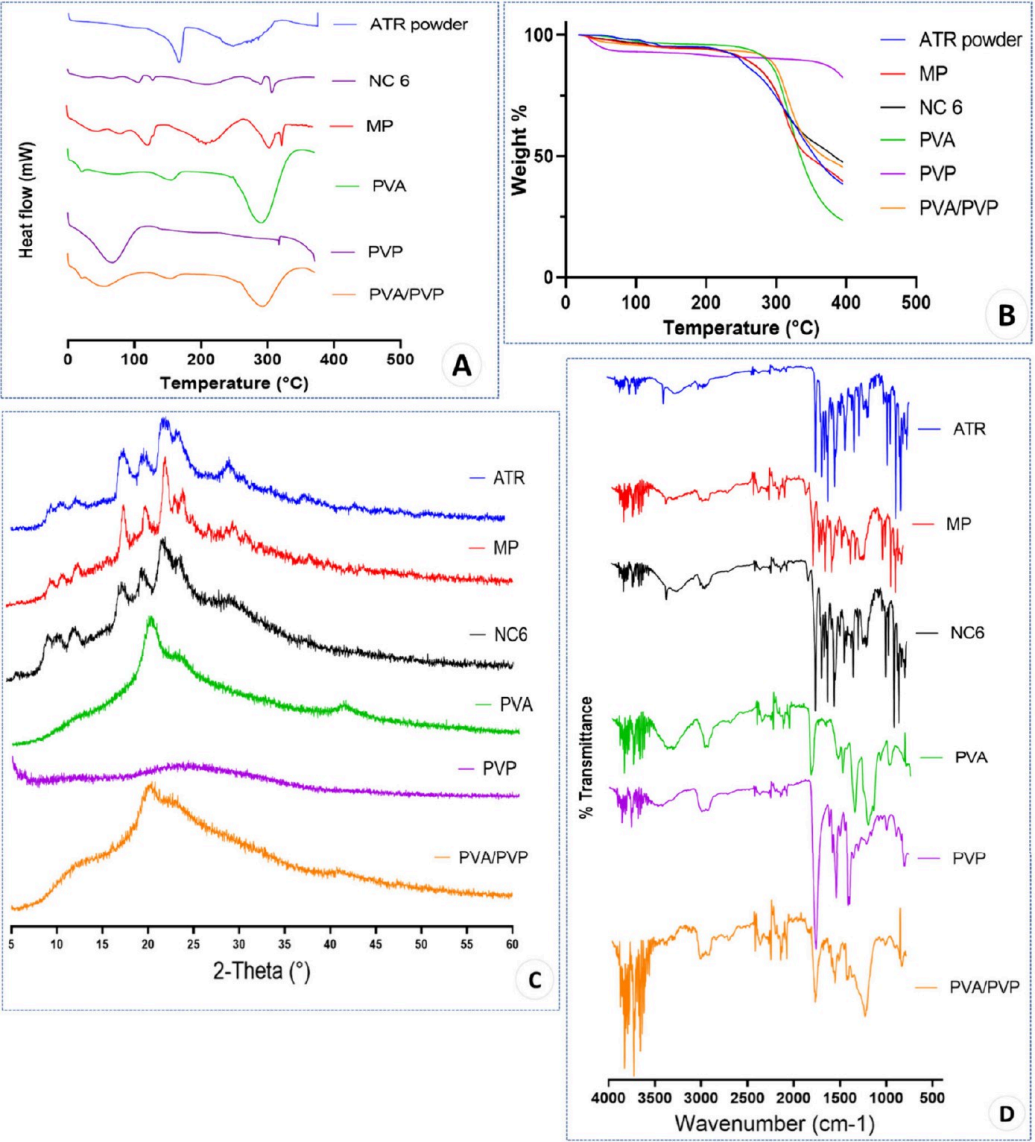
Representative (A) DSC thermograms, (B) TGA thermograms, (C) PXRD diffractograms and (D) FTIR spectra of the pure ATR powder, MP formulation, NC 6, PVA, PVP and PVA/PVP mixture in a 1:1 ratio.

The percentage weight loss with increasing temperature in the DSC profiles (Figure 9A) exhibited a very similar pattern between pure ATR and its incorporation into the MP and NC formulations. An endothermic peak was observed for the crude ATR powder at approximately 158.4 °C, which corresponded to the reported melting point of ATR of 159.14 °C.^60^ This endothermic peak diminished and shifted slightly in the ATR MP and NC formulations to 148.4 and 132.69 °C, respectively. Hence, a near disappearance of the melting peak and a slight shift to the left were observed in this thermogram. This could be ascribed to a partial amorphization that might have occurred due to heating during the experiment (loss of melting event). The endothermic peaks in the thermograms of PVP at 93.13 °C and PVA/PVP at 80.83 °C could be attributed to the early melting of the stabilizers. This could also be due to the partial amorphization of the drug during analysis. The endothermic peak at 132.69 °C indicated that the melting point of ATR in NC 6 was lower than that of both pure ATR and the MPs, which could be due to the lower amount of powder loaded into the pan as a result of the porous nature of the lyophilized NCs.

TGA thermograms revealed comparable results, with both the NC 6 and MP formulations exhibiting patterns similar to those of pure ATR powder (Figure 9B). This confirms the lack of interactions between ATR and PVA/PVP when they are incorporated in the formulation. Before reaching 100 °C, a minor weight reduction of less than 5% was observed, which was most likely due to the evaporation of adsorbed water molecules on the formulations’ surfaces. The compounds then began to rapidly lose weight after 300 °C, indicating that their structure had started to thermally decompose. The loss of three water molecules during crystallization was indicated by the distinct water loss observed in the 257–271 °C region of the ATR TGA curves.^61^ This water loss can be attributed to the intrinsic structure of the ATR.

As shown in Figure 9C, the PXRD patterns indicated the crystallinity of ATR both before and after the incorporation of PVA/PVP into the formulations. This suggests that there were no phase shifts or chemical interactions between pure ATR and the two lead formulations. This also implies that there were no significant phase changes or chemical reactions between ATR and the excipients in either formulation. In contrast, the PXRD pattern of the PVA/PVP mixture showed a single broad peak, potentially indicating that both polymers exhibit amorphous properties.^62^

ATR-FTIR analysis revealed that there were no chemical interactions between ATR and the excipients in either the MP or NC formulations. This was demonstrated by the presence of the characteristic bands of ATR in the spectra of both the MP and NC formulations, as depicted in Figure 9D.

### 3.5 Actual ATR Content in D-MAPs

**Figure 10:**
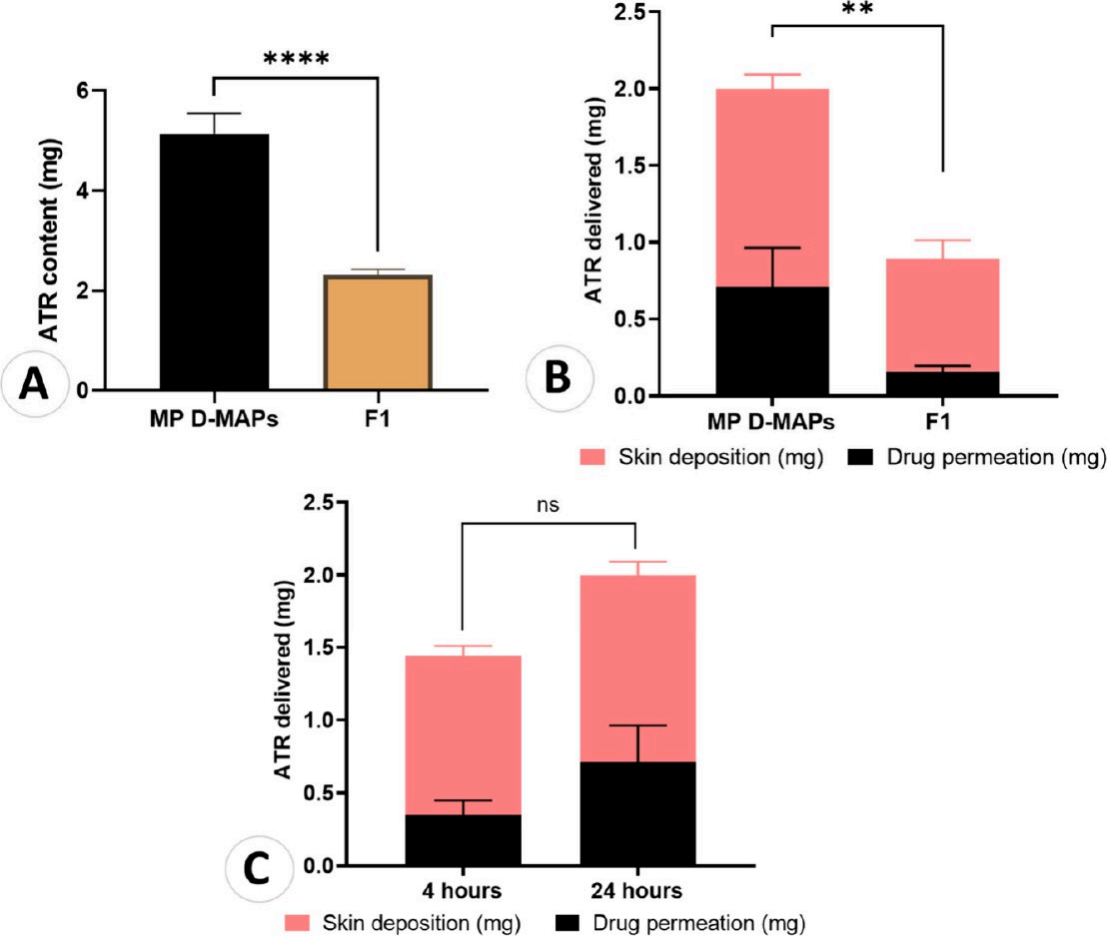
(A) ATR content in both MP-loaded D-MAPs and F1 D-MAPs. (Means + SDs, *n* = 4). (****p < 0.0001). (B) The quantity of ATR in mg recovered from each compartment following the application of MP D-MAPs and F1 for 24 h. (Means + SDs, *n* = 3). (**p = 0.0059). (C) The quantity of ATR in mg recovered from each compartment following the application of MP-loaded D-MAPs for 4 h compared to 24 h wear time, showing no significant difference in the cumulative amount delivered from both setups. (Means + SDs, *n* = 3).

The actual ATR percentage in NC 6 was estimated to be 67.21 ± 1.97%, which aligns with the previously estimated theoretical ATR percentage of 71.4%. The actual ATR content in both the MP-loaded D-MAPs and F1 D-MAPs was evaluated, and the results are shown in Figure 10A below. The ATR content of MP D-MAPs (5.15 ± 0.40 mg) was significantly greater than that of F1 D-MAPs (2.37 ± 0.04 mg) (*p* < 0.0001). This finding suggested that approximately twice as much ATR could be loaded into MP D-MAPs as into F1 D-MAPs.

### 3.6 Ex Vivo Skin Deposition Studies

*Ex vivo* skin deposition studies were performed on full-thickness neonatal porcine skin to evaluate the total amount of ATR delivered to the receiver compartment of Franz cells at 24 h, 4 h, or both time points. Both MP D-MAPs and F1 (NC 6-loaded D-MAPs) formulations were studied in this context due to their superior mechanical characteristics. After 24 h of MP D-MAP application, a mean of 1.3 ± 0.1 mg of ATR was extracted from the skin, while 0.7 ± 0.7 mg was extracted from the receiver compartment. Consequently, 2.02 ± 0.35 mg of ATR was delivered in total to both compartments, accounting for approximately 39.29 ± 6.7% of the ATR that was initially loaded into the D-MAPs. On the other hand, the average amount of ATR extracted from the skin and receiver compartments was 0.75 ± 0.1 mg and 0.2 ± 0.06 mg, respectively, following the application of F1 for 24 h. This translates to a total ATR delivery of approximately 0.89 ± 0.12 mg into both compartments, representing approximately 38.42 ± 5.13% of the ATR amount initially loaded into F1 D-MAPs. Although both MP D-MAPs and F1 had similar percentages of ATR deposited in the skin and receiver compartments (39.29 ± 6.7% and 38.42 ± 5.13%, respectively), there was a significant difference in the quantity delivered. When compared to F1, MP-loaded D-MAPs could significantly deliver and deposit greater quantities of ATR (*p* = 0.0059). These findings are summarized in Figure 10B below.

At 24 h after application, the delivery of ATR into the skin and receiver compartments was significantly greater for the MP D-MAPs (*p* < 0.05), likely due to their higher drug content. As a result, they were evaluated again after 4 h of skin deposition. The results revealed that with an average of 1.1 ± 0.1 mg of ATR was recovered from the skin, while 0.3 ± 0.1 mg was recovered from the receiver compartment. As shown in Figure 10C, the total amount of ATR recovered from both compartments was 1.44 ± 0.15 mg, representing approximately 28.03 ± 0.79% of the ATR initially loaded into the D-MAPs. At 4 or 24 h, there was no significant difference in the cumulative quantity of ATR delivered to both compartments (*p* > 0.05). This finding implies that the wear time of D-MAPs with this formulation might be shortened to 4 h rather than 24 h. However, due to the *in vivo* study restrictions and regulations, this could not be assessed in the current study, but could be a perspective to investigate in future studies.

D-MAPs from both formulations (MP-loaded and F1) showed full insertion into the skin and complete separation from the backing layer upon removal from the skin; there were no apparent ATR residues in the backing layer. Concerns regarding the back migration of ATR from the tips into the backing layer were not applicable in this case, given the hydrophilic composition of the backing layer (primarily PVP and water) and the hydrophobic nature of ATR (aqueous solubility of only 0.1 mg/mL^7,63^). Conversely, as shown in Figure S4 in the Supporting Information, the appearance of white tips within the skin following D-MAP removal verified the complete insertion of the D-MAPs and the deposition of their cargo into the skin.

The distinct bilayer casting method employed in this formulation, in which ATR was solely loaded into the MN arrays before a drug-free baseplate was cast, also likely had a significant impact on the tip separation from the baseplate. At the application site, there was a discernible implantation of dissolving tips containing ATR (white in color) after the removal of D-MAP. Some white residues of the ATR-containing formulations were seen on the surface of the skin. This could be because the drug was loaded into the entire length of the MN tips, although it is unlikely to be harmful. Prospective studies may look exclusively into loading drug-containing formulations into D-MAPs’ array tips to address this issue. Given that approximately 70% of the needle heights were successfully inserted both *in vitro* and *ex vivo*, it may be possible to reduce drug accumulation on the skin by loading a drug-containing formulation into approximately 50% of the length of the MN array. By delivering the full dose of the drug within the skin, this adaptation may improve delivery rates, even though the overall amount delivered may not change significantly.

### 3.7 In Vivo Depot Delivery of ATR from Sprague–Dawley Rats

#### 3.7.1 In Vivo Delivery of ATR Using D-MAPs

An *in vivo* study was conducted to determine the transferability of ATR MP-loaded D-MAPs from an *ex vivo* model to an *in vivo* study setup. ATR pharmacokinetics were assessed for 14 days following a single dose administration to the rats on day 0. Each rat received 4 D-MAPs at a total dose of approximately 20 mg/rat. Some rats showed mild erythema at the application site following D-MAPs removal (Figure S4A in the Supporting Information), which was completely resolved and healed within 1 h, aligning with the findings in earlier reports.^21,64^

ATR-containing dissolving tips, which are white in color, were visibly implanted at the D-MAP application site (Figure S5A-B). At 24 h, when the D-MAPs were removed, the MNs arrays (the part containing ATR) were deposited in the skin and were completely separated from the baseplate, which was almost entirely dissolved (Figure S5A-C).

Given that ATR was loaded throughout the entire length of the MN array rather than just the tips, some drug was found on the skin surface after the D-MAPs removal (Figure S5A-B). This is not harmful, and it could be avoided by loading the drug only in the array tips.

**Figure 11:**
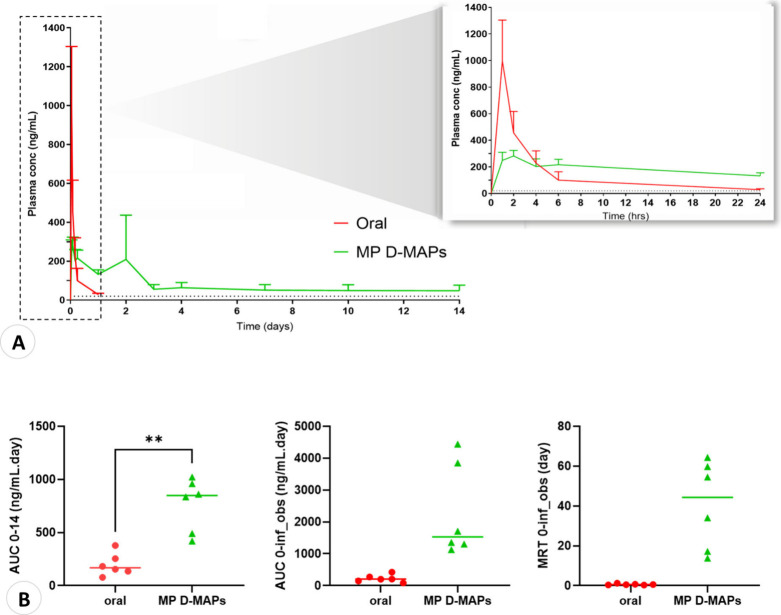
(A) The plasma concentration of the ATR following the administration of the MP D-MAPs compared to the oral control adapted from Naser et al.^1^ ATR plasma profile of the rats given 4 MP D-MAPs each following the in vivo study for 14 days. (Means + SDs, *n* = 3 at 1, 2, 4, and 6 h, *n* = 6 at all other time points). The LOQ of the analytical method (20 ng/mL) is denoted by the black dashed line. This threshold also corresponds to the therapeutic dose of ATR in rats over a 6-h period after the oral administration of ATR.^67^ Additionally, the orange dashed line represents the highest plasma concentration of ATR (10 ng/mL) observed after the administration of 10 mg/day ATR for 14 days in patients recently diagnosed with hyperlipidemia.^65^ It also has the highest plasma concentration following the administration of 10, 20, or 40 mg of ATR in grapefruit juice over a 90-day period.^66^ (B) Statistical analysis of the AUC _0–14_, AUC _0-inf_obs_, and MRT_0-inf_obs_ in rats between the MP D-MAPs cohort and the control group adapted from Naser et al.^1^

As previously reported by the authors in 2023,^1^ ATR was given in an oral control cohort compared to another type of MAPs, namely hydrogel-forming MAPs. To reduce the number of the animals used in compliance with the 3Rs principle (replacement, reduction, and refinement), the control cohort was not repeated in this study. However, the comparison between the MP D-MAPs results reported in this work and the control cohort reported by us in our previous study^1^ are summarized in Figure 11 and Table 6. Figure 11 A depicts the *in vivo* plasma profile of ATR in rats receiving D-MAPs in comparison with that of the oral control group. Within the first hour, a rapid increase in the plasma levels of ATR was noted (248.57 ± 61.1 ng/mL). Nonetheless, the *T*_max_ was only reached at t = 2 h when *C*_max_ was 281.97 ± 41.73 ng/mL. Over the first 2 days, ATR levels remained at a comparable level. On the third day, ATR plasma levels declined to 55.57 ± 23.71 ng/mL, and they remained relatively constant until the end of the 14-day study, at a concentration of 47.91 ± 29.1 ng/mL.

**Table 6:** In Vivo Plasma Pharmacokinetic Parameters of ATR after Oral and MP D-MAPs Administration to Sprague–Dawley Rats^a^

Parameter	Unit	Control (oral)	D-MAPs
*T*_max_	Hour	1	2
*C*_max_	ng/mL	998.2 ± 306.3	248.57 ± 61.1
AUC_0-t_	ng/mL·day	197.05 ± 106.6	764.61 ± 249.84
AUC_0-inf_obs_	ng/mL·day	222.97 ± 116.45	2296.80 ± 1455.81
MRT_0-inf_obs_	Day	0.61 ± 0.35	40.60 ± 22.07

a Means ± SDs, *n* = 6 for each group.

Throughout the course of the 14-day study period, therapeutically relevant plasma concentrations of ATR were attained within the first hour of D-MAP application,^65,66^ and were maintained throughout the study. This novel finding proposes the potential depot delivery of ATR across the skin, allowing for the sustained release of a therapeutic dose of the compound over a period of 2 weeks following a single dose administration of MP D-MAPs. Therefore, to the best of the authors’ knowledge, this is the first report of long-acting delivery of ATR using D-MAPs loaded with ATR MPs.

#### 3.7.2 Pharmacokinetic Analysis of ATR and Relative Bioavailability

Compared to those in the oral group, the overall pharmacokinetic profiles of plasma ATR were significantly improved using D-MAP formulations, as shown in Table 6. The AUC _0–14_ of the D-MAP group (764.62 ± 49.84 ng/mL/day) was significantly (*p* < 0.05) greater than the AUC _0–14_ of the oral group (197.05 ± 106.6 ng/mL/day).

Despite the greater AUC_0-inf_obs_ in the D-MAPs group (2296.80 ± 1455.81 ng/mL·day) compared to the oral control (222.97 ± 116.45 ng/mL·day), the difference was not statistically significant. Similar findings were also observed for MRT, where the D-MAPs cohort attained significantly higher values (*p* < 0.05) of 40.60 ± 22.07 days, in contrast to the control group possessing an MRT of 0.61 ± 0.35 days. Interestingly, a delay in *T*_max_ was noted in the D-MAPs group, indicating a possible long-acting effect compared to the oral route.

Based on this information, the rats in the control group received approximately 1.4 mg of ATR over 14 days. Using the previously described eq 6, the transdermal bioavailability of ATR using D-MAPs (F_D-MAPs_) was calculated to be 0.27%. Therefore, approximately 5.4 mg of ATR was delivered to the rats via D-MAPs throughout the study.

#### 3.7.3 Estimation of Human Patch Size

The data were thoroughly analyzed to determine the approximate patch size. The oral bioavailability of ATR is approximately 14% when it is taken orally at doses ranging from 10 to 80 mg/day.^3,67,68^ Therefore, when employing D-MAPs, 5.6 mg of ATR must be transdermally delivered to reach a dosage equal to 40 mg/day administered orally in humans. Considering that approximately 4.82 mg of ATR was delivered from 4 different D-MAPs with a total combined area of 3 cm^2^, the required patch size to deliver a daily dose of 40 mg/day was estimated to be 3.1 cm^2^. As a result, the patch size of D-MAPs required to provide a dose comparable to 40 mg/day to humans over 14 days after a single application for 24 h would be 43.3 cm^2^.

In our previous study, Naser et al.^1^ showed that ATR could also be intradermally deposited following the application of hydrogel-forming MAPs combined with a PEG-based solid dispersion reservoir. In that study, the patch size needed to deliver therapeutically relevant human doses of ATR was estimated to be 32.5 cm^2^. Both patch sizes, as previously reported by^1^ and as obtained in this investigation employing D-MAPs, are within the acceptable limits of commercially available transdermal patch sizes. Capsaicin transdermal patches, for example, used for the topical treatment of neuropathic pain, can be as large as 280 cm^2^.^69^ Furthermore, the area of regularly applied fentanyl-loaded transdermal patches is approximately 42 cm,^2,69^ which is similar to the anticipated size for D-MAPs. Therefore, this finding emphasizes the efficiency of translating the use of MAPs in the long-acting delivery of ATR to a clinical setup in future investigations. Due to reduced dosing frequencies, long-acting ATR delivery via D-MAPs has the potential to improve patient quality of life and treatment adherence.^70,71^ Consequently, MAPs hold promise for improving patient outcomes through pain-free delivery that maximizes therapeutic effects over the long-term.

## 4 Conclusion

This study describes the production of MP- and NC-loaded D-MAPs for efficient transdermal delivery of the hydrophobic drug ATR. The formulations were developed using relatively straightforward techniques that can be easily scalable to manufacturing levels. ATR MPs were generated using SpeedMixer in a simple mixing step, whereas nanocrystals were created using a laboratory-scale wet bead-milling strategy, yielding stable particles within a size range of 150–250 nm. The mechanical properties, drug loading, and *in vitro* insertion efficiency of both MP-loaded and NC-loaded D-MAPs were assessed. MP D-MAPs and F1 NC D-MAPs were chosen for additional investigations, considering their homogeneity, drug-carrying capacity, and favorable mechanical properties. The superior performance of MP D-MAPs in terms of drug loading and deposition into full-thickness excised neonatal porcine skin was established in subsequent *ex vivo* skin deposition studies.

In addition, the nearly identical *in vitro* release profiles of both formulations led to the selection of MP D-MAPs for an unprecedented *in vivo* study. Over a two-week period, this study demonstrated the efficacy of MP D-MAPs for long-acting ATR delivery. The novel technology suggested in this study provides a promising, minimally invasive substitute for long-acting ATR delivery, with prospective advantages for patient acceptance, compliance, and therapeutic outcomes.

Future research avenues may involve pharmacodynamic studies on animals to further explore the correlation between drug delivery and therapeutic efficiency. Furthermore, regulatory requirements, particularly during the production stage, whether an aseptic production process will be required or only an end-product sterilization would be sufficient. This is mainly to ensure the production of this innovative system on a large scale, while ensuring a cost-effective production approach.

## Supporting Information Available

Additional experimental details, materials, and methods, including photographs of experimental setup; Fabrication of high-density molds; Fabrication of ATR NCs using wet bead milling technique; The effect of the amount of ATR added into each NC formulation, along with the type and volume of stabilizer on the PS, PDI, and ZP of the final NCs; Lyophilization of NCs; In vitro release study suding dialysis membranes; Ex vivo skin deposition studies of ATR; In vivo delivery of ATR images; Pharmaceutical analysis of ATR (PDF)
