# Supplementary material for: Atorvastatin-Loaded Dissolving Microarray Patches for Long-Acting Microdepot Delivery: Comparison of Nanoparticle and Microparticle Drug Formulations
Source: ACS Appl Mater Interfaces. 2024 Oct 2;16(41):55027–44. doi: 10.1021/acsami.4c05517 (PMC11492242; doi:10.1021/acsami.4c05517)
Supplement: Supplementary file 1 — am4c05517_si_001.pdf [file am4c05517_si_001.pdf]

Atorvastatin-Loaded Dissolving Microarray Patches for Long-acting Microdepot Delivery: Comparison of Nanoparticle and Microparticle Drug Formulations

*Yara A. Naser [1], Lalitkumar K. Vora [1], Ismaiel A. Tekko [1,2], Ke Peng [1], Fabiana Volpe-Zanutto [1], Brett Greer [3], Alejandro Paredes[1], Helen O. McCarthy [1], Ryan F. Donnelly [1]**

[1]School of Pharmacy, Queen's University Belfast, Medical Biology Centre, 97 Lisburn Road, Belfast, BT9 7BL, Northern Ireland, UK.

[2]Department of Pharmaceutics and Pharmaceutical Technology, Faculty of Pharmacy, Aleppo University, Aleppo, Syria.

[3]Institute for Global Food Security, School of Biological Science, Queen's University Belfast, 19 Chlorine Gardens, Belfast BT9 5DL, Northern Ireland, UK.

***Corresponding author:**

Professor Ryan F. Donnelly

Chair in Pharmaceutical Technology

School of Pharmacy - Queen's University Belfast

Medical Biology Centre

97 Lisburn Road

Belfast

BT9 7BL, Northern Ireland, United Kingdom

Tel: +44 (0) 28 90 972 251

Fax: +44 (0) 28 90 247 794

Email: r.donnelly@qub.ac.uk

Supporting information:

1. Fabrication of high-density moulds

To create the moulds, Xiameter® silicone base (white) and its green curing agent were thoroughly mixed in a 10:1 w/w ratio. The blends underwent centrifugation at 5,000 rpm for 10 minutes. The resulting formulation was cast into 3D printed templates, following a previously reported method [1]. After tapping the templates to eliminate air bubbles, they were allowed to cure overnight at room temperature. Subsequently, the moulds were detached from the 3D printed holders, and flat micromould sheets, industrially manufactured through injection moulding, were affixed to the flat surface of the moulds.

These high-density MAPs sheets featured 600 pyramidal needles within a 0.75 cm^2 area, with a base measuring 300 x 300 µm, array heights of 750 µm, and interspacing of 50 µm. The sheets were attached to the moulds using a small amount of transparent LSR9-9508-30 silicone elastomer mixture in a 1:1 w/w ratio of Part A to Part B. The moulds were then cured at $80^{o}C$ for 20 minutes, rendering them ready for use.

2. Fabrication of ATR NCs using wet bead milling technique

The parameters which were thoroughly investigated and explored to generate various formulations are summarised in Table S 1. Notably, the ATR amount, stabilizer type, and stabilizer volume were purposefully changed to obtain the required particle size range (150 - 250 nm), whereas other parameters remained constant throughout the experiment.

Table S 1. The different NC formulations investigated, and the parameters used in the preparation of each NC formulation.

NC	ATR (mg)	Stabilizer used		Magnet stirrers (12x6mm)	Beads		Milling time (h)
		Type	Vol. (mL)		Size (mm)	Vol. (mL)	
**NC 1**	100	2% PVA	6	4	0.1-0.2	2	21
**NC 2**	200	2% PVA	6	4	0.1-0.2	2	21
**NC 3**	300	2% PVA	6	4	0.1-0.2	2	21
**NC 4**	100	2% PVA/PVP	6	4	0.1-0.2	2	21
**NC 5**	200	2% PVA/PVP	6	4	0.1-0.2	2	21
**NC 6**	300	2% PVA/PVP	6	4	0.1-0.2	2	21
**NC 7**	400	2% PVA/PVP	5	4	0.1-0.2	2	21
**NC 8**	400	2% PVA	5	4	0.1-0.2	2	21
**NC 9**	100	2% PVP	6	4	0.1-0.2	2	21
**NC 10**	200	2% PVP	6	4	0.1-0.2	2	21

The effect of the amount of ATR added into each NC formulation, along with the type and volume of stabiliser on the PS, PDI, and ZP of the final NCs were studied and investigated before and after lyophilization. The results for the NS prelyophilization are shown in Figure S 1, whereas the results after lyophilization are summarized in Figure S 2.

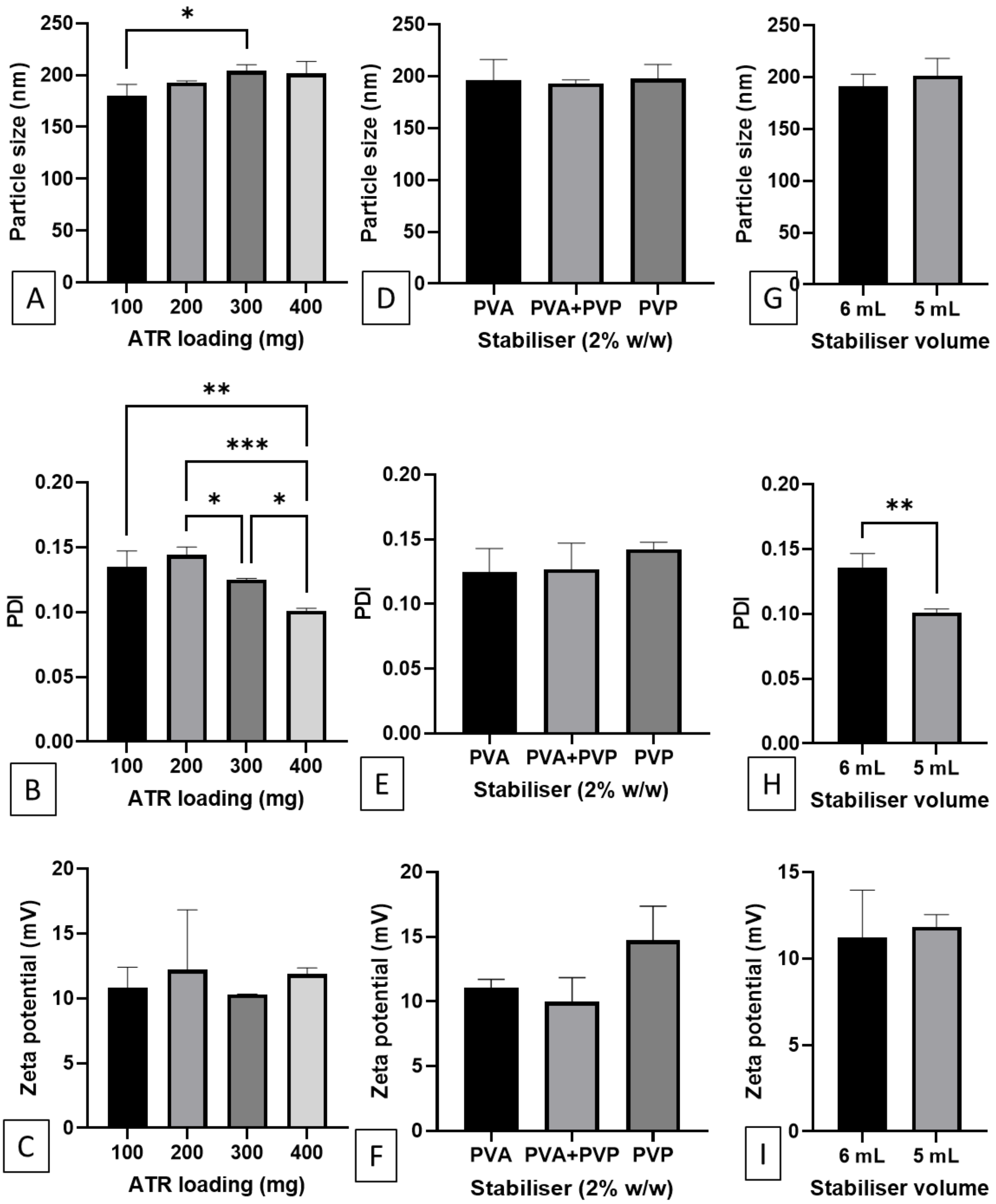

Figure S 1.. Results showing the effect of the drug loading on (A) particle size, (B) polydispersity index and (C) zeta potential. Additionally, the effect of the stabilizer type on (D) particle size, (E) polydispersity index and (F) zeta potential. Along with the effect of the stabiliser volume on (G) particle size, (H) polydispersity index and (I) zeta potential, of the nanosuspensions prior to their lyophilisation. (Means + SD, n≥ 3). **p* < 0.037, ***p* = 0.001 ****p* = 0.0003.

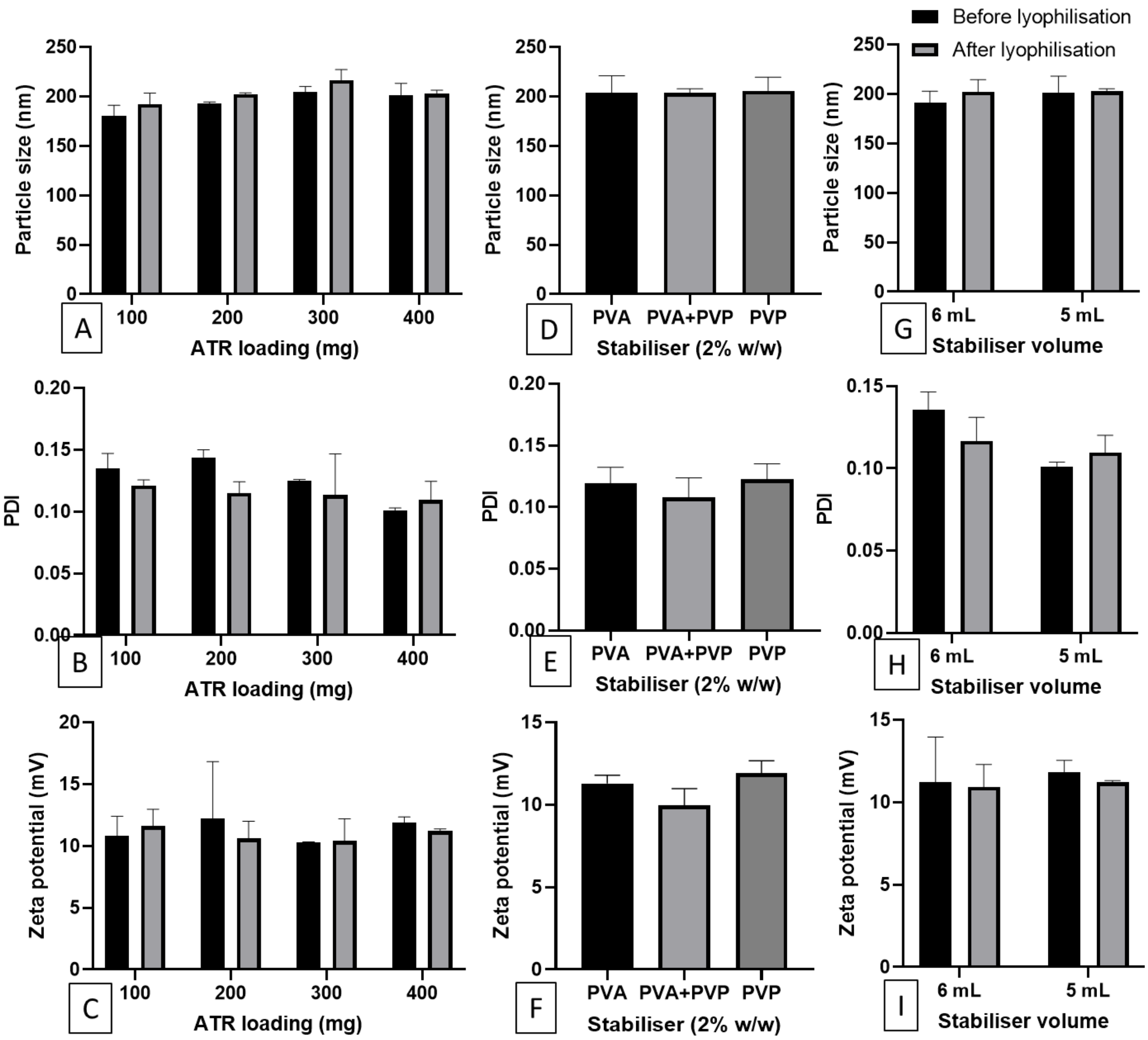

Figure S 2. Results showing the effect of the drug loading on (A) particle size, (B) polydispersity index and (C) zeta potential. Additionally, the effect of the stabilizer type on (D) particle size, (E) polydispersity index and (F) zeta potential. Along with the effect of the stabiliser volume on (G) particle size, (H) polydispersity index and (I) zeta potential, of the nanosuspension before and after their lyophilisation using the freeze dryer. (Means + SD, n ≥ 3).

3. Lyophilization of NCs

Table S 2. The parameters of the cycle employed in the lyophilisation of the nanosuspensions.

Temperature (°C)	Time (mins)	Set
-40	30	Ramp
-40	60	Hold
-30	30	Ramp
-30	60	Hold
-20	30	Ramp
-20	60	Hold
-10	30	Ramp
-10	500	Hold
0	30	Ramp
10	60	Ramp
25	60	Ramp
25	600	Hold

4. *In vitro* release study using dialysis membrane

a. Method:

The *in vitro* release of ATR was investigated using a dialysis membrane method as previously detailed [2]. The release media used was 1% w/v SLS in PBS (pH 7.4) to maintain sink conditions. Two lead formulations were examined, MP D-MAPs and the lead NC D-MAPs. The required amounts of ATR, PVA and PVP were weighed out, then dispersed in 1% w/v SLS in PBS. The same was done for ATR-loaded NCs. The dispersions were then placed in the Spectra-Por®, MWCO 12-14 kDa dialysis membranes. Afterwards, they were clamped to prevent the leakage of the dispersion into the release media. They were immediately placed individually in bottles containing 100 mL of the release media, and were put in an orbital shaker at 100 rpm and 37°C. At predefined timepoints of 0.25, 0.5, 1, 2, 3, 4, 5, 6, 8, 24, 48, 72 and 96 hours (over 4 days), 1 mL of each bottle was sampled and replaced with fresh release media. Samples were appropriately diluted when necessary prior to their HPLC analysis to quantify the amount of ATR released.

b. Results and discussion:

The *in vitro* release profiles of NC 6 and ATR-loaded MP formulation are presented Figure S 3. Over the course of 4 days, 92.34 ± 11.23% and 92.98 ± 2.2% of ATR initially loaded was eventually released from ATR-loaded MP and NC 6 formulations, respectively. Based on that, no significant difference was denoted in the percentage of drug released over time from both MP and NC 6 formulations ($p > 0.05$). Based on all previous results, MP-loaded D-MAPs was to be brought forward for further *in vivo* studies rather than the NC-loaded formulation. for multiple reasons. Firstly, and despite the fact that NC were fabricated in a simple way, the fabrication of MP D-MAPs was more straightforward, during the mixing of the polymers with ATR. This renders the method to be easily scalable to an industrial set up, more cost effective and less time-consuming. Moreover, ATR loading in MP D-MAPs was significantly higher than the lead NC D-MAPs formulation ($p < 0.05$), resulting in significantly higher ($p < 0.05$) amounts of ATR deposited in the skin following the *ex vivo* studies. Furthermore, MP D-MAPs possessed a favourable insertion profile and sufficient mechanical strength. Additionally, the release profile of ATR from both MP and NC loaded formulation followed a very similar general trend. This could suggest that ATR-loaded MP formulation possessed all the desired characteristics and is also more straightforward and cost-effective for upscaling to an industrial level.

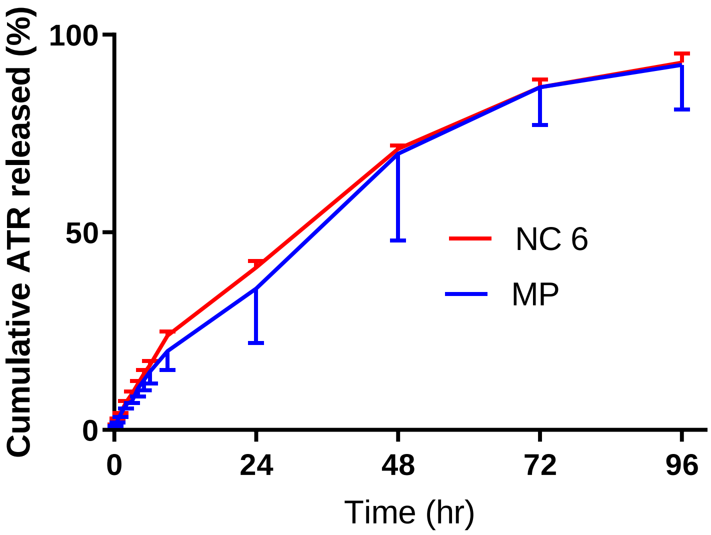

Figure S 3. *In vitro* release profiles of ATR-loaded MP formulation and NC 6 over 96 hours from the dialysis membranes. (Means ± SD, n=3).

5. Ex vivo skin deposition studies of ATR

Microscopic images of the skin prior to and following the removal of the excess ATR are presented in Figure S 4 below.

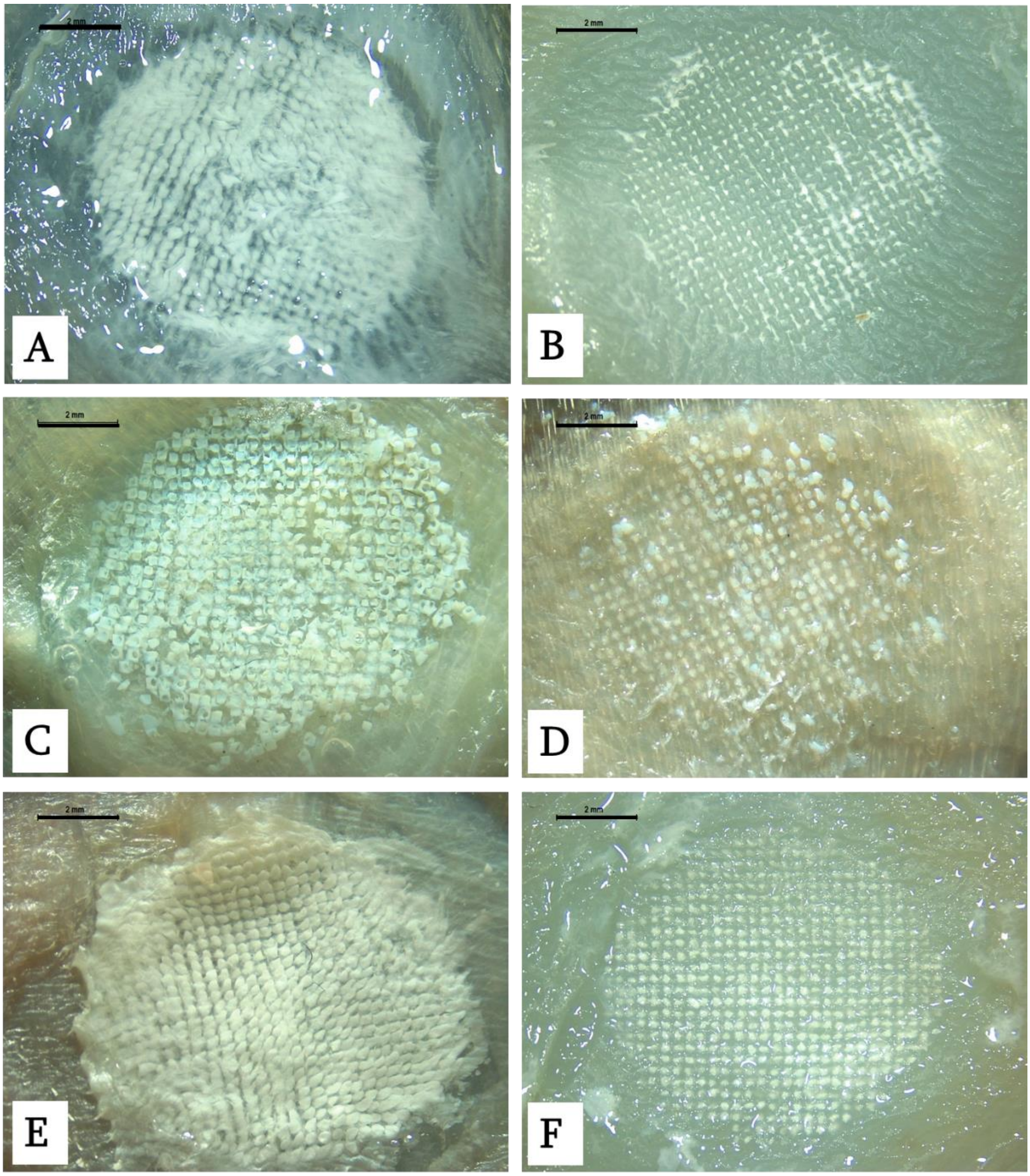

Figure S 4. Excised neonatal porcine skin 24 hours after concluding the ex vivo skin deposition experiment following the application of MP-DMAPs (A) before and (B) after removing the excess ATR. The skin following the application of F1 D-MAPs, (C) prior to and (D) following the removal of excess ATR from the surface. On the other hand, (E) shows the skin 4 hours following the application of MP-DMAPs before and (F) after removing the excess ATR. Scale bars in all images correspond to 2 mm in length.

6. In vivo delivery of ATR

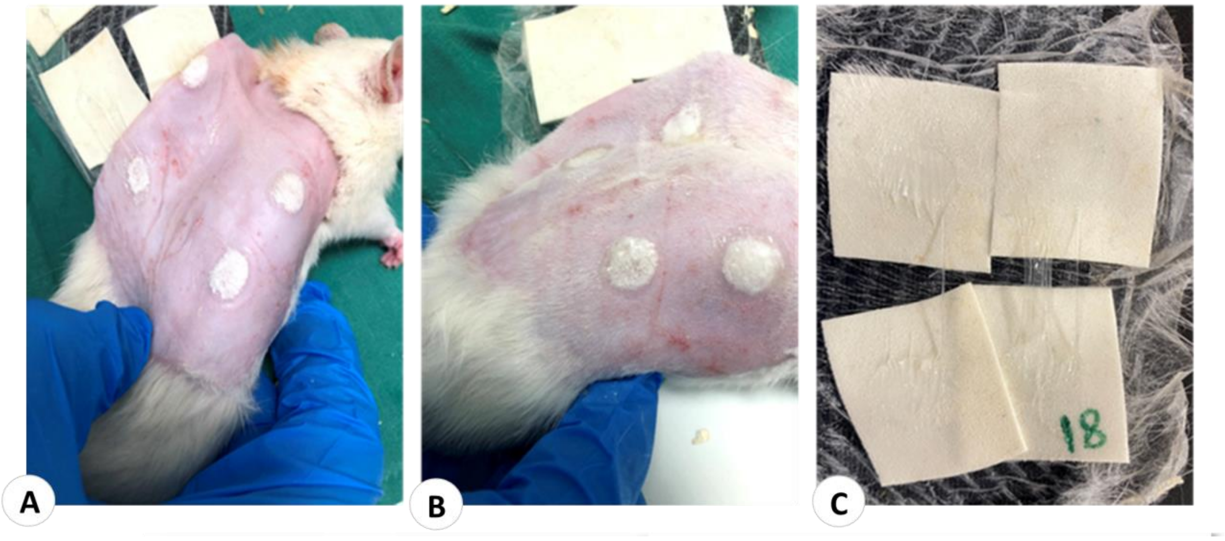

Figure S 5. (A) The back area of a rat after removal of the adhesive layer following the application of D-MAPs. **(B)** The site of D-MAP application showing tip implantation and the deposition of ATR into the dorsal skin of the rats. **(C)** The backing adhesive layer used on top of D-MAPs.

7. Pharmaceutical analysis of ATR

The quantification of ATR was performed as previously described by Naser et al. [39]. The details of the method are summarized in Table S 3 below.

Table S 3. Summary of the analytical and bioanalytical method used for the analysis of ATR.

Method	Analytical method	Bioanalytical method
**Machine**	RP-HPLC system, specifically the Agilent 1220 series	ACQUITY Ultra-Performance Liquid Chromatography (UPLC) i-Class system coupled with a Xevo TQ-MS (triple quadrupole MS/MS) mass spectrometer
**Sample matrix**	1%w/v SLS in PBS (based on a preliminary saturation solubility study)	Rat plasma
**Detector**	UV detection at 240 nm	TQ-MS mass spectrometer using MRM detection, precursor ion 559.45 *m/z* and base fragment ion 446.35 *m/z*
**Column**	Phenomenex SphereClone® C-18 (ODS1) column: 150 mm x 4.6 mm internal diameter, 5 µm packing	Zorbax Eclipse XDB C-18 column: 50 mm x 4.6 mm internal diameter, 1.8 µm packing
**Mobile phase composition**	Isocratic method:<br>Phosphoric acid solution at pH=2.1 (A) and methanol (B), 40%: 60% v/v	Gradient method:<br>0.1% v/v formic acid in water at pH 2.1 (A), and acetonitrile (B):<br>30% A: 70% B (0-2.5 min)<br>5% A: 95% B (2.6-3.6 min)<br>30% A: 70% B (3.6-5 min)
**Range**	0.125-50 $\mu g.mL^{-1}$	20-1000 $ng.mL^{-1}$
**Flow rate (mL/min)**	1	0.5
**Injection volume (µL)**	30	5
**Retention time**	5.2 min	1.54 min
**LOQ**	1 $\mu g.mL^{-1}$	20 $ng.mL^{-1}$

References:

1. Ramöller I, McAlister E, Bogan A, Cordeiro A, Donnelly R: **Novel design approaches in the fabrication of polymeric microarray patches *via* micromoulding.** *Micromachines* 2020, **11:**554.
2. Jain AK, Swarnakar NK, Godugu C, Singh RP, Jain S: **The effect of the oral administration of polymeric nanoparticles on the efficacy and toxicity of tamoxifen.** *Biomaterials* 2011, **32:**503-515.
